# Assessing the informative value of macroeconomic indicators for public health forecasting

**DOI:** 10.1371/journal.pdig.0001599

**Published:** 2026-08-20

**Authors:** Shome Chakraborty, Fardil Khan, Soutik Ghosal

**Affiliations:** 1 Gabelli School of Business, Fordham University, New York, New York, United States of America; 2 Harpur College of Arts and Sciences, Binghamton University, Binghamton, New York, United States of America; 3 Public Health Sciences Department, School of Medicine, University of Virginia, Charlottesville, Virginia, United States of America; Yonsei University College of Medicine, KOREA, REPUBLIC OF

## Abstract

Macroeconomic conditions influence the environments in which health systems operate, yet their value as leading signals of health-system capacity has not been systematically evaluated. In this study, we examined whether certain macroeconomic indicators contained predictive information for several capacity-related public health targets in the United States: employment in the health and social assistance workforce, new business applications in the sector, and health care construction spending. Using seasonally adjusted monthly time-series data collected from government sources, we evaluated multiple forecasting approaches—including neural network models with different optimization strategies, generalized additive models, random forests, and time series models with exogenous macroeconomic indicators—under different model fitting designs. Across the evaluation settings, we found that macroeconomic indicators are associated with improved predictive performance for some public health targets—particularly workforce measures—while other targets exhibit weaker or less stable predictability. Models emphasizing stability and implicit regularization tend to perform more reliably during periods of economic volatility. These findings suggest that macroeconomic indicators may serve as useful upstream signals for digital public health monitoring, while underscoring the need for careful model selection and validation when translating economic trends into health-system forecasting tools.

## Author summary

Public health systems are affected not only by medical events, but also by changes in the economy—such as shifts in employment, business activity, and investment. These economic indicators are tracked every month and are often available before changes in the health system become visible. In this study, we asked whether these economic trends can help anticipate future changes in health-system capacity, such as workforce levels and infrastructure investment. We tested several types of forecasting models and evaluation approaches to see whether the results were consistent across methods and time periods. We found that some health-system measures showed clear and reliable links with macroeconomic trends, while others were much harder to predict. Our findings suggest that economic data may serve as one useful early-signal source for public health monitoring, but they also highlight the need for caution and careful validation before such predictions are used in real-world decision-making.

## Introduction

### Macroeconomic signals and population health

The chronic under-funding and fragmentation of public health infrastructure in the United States and globally have left health systems increasingly vulnerable to macroeconomic shocks [1,2]. From pandemic-induced supply chain failures to inflation-driven labor shortages, systemic stress often originates far upstream of clinical settings, within economic conditions that destabilize health system operations well before visible breakdowns occur. These disruptions disproportionately affect low-resource settings, where even modest fiscal shocks can cascade into appointment bottlenecks, stockouts of essential drugs, emergency department crowding, and delayed preventive care [3].

Macroeconomic indicators such as retail activity, trade volume, inventory levels, and business formation capture these upstream pressures. Although such indicators are traditionally monitored for fiscal or monetary policy, they also signal downstream constraints on healthcare delivery, workforce stability, and access to care [4]. A contraction in consumer demand, for example, may precede reductions in employer-sponsored insurance coverage or delays in healthcare utilization, while disruptions in trade and logistics can directly affect medical supply chains [5]. Despite these connections, macroeconomic indicators remain underutilized in population health forecasting frameworks, particularly in systematic, model-based evaluations. In this study, we focus on three public health targets that reflect key dimensions of health system capacity: workforce (health care and social assistance employment), organizational activity (health care business applications), and infrastructure investment (health care construction spending). These measures serve as proxies for health system strain, capturing complementary aspects of labor availability, service expansion, and capital investment.

### Digital data streams for public health forecasting

The growing availability of high-frequency, digitally collected economic data has created new opportunities for population-level health surveillance. At the same time, advances in machine learning (ML) have expanded the analytical toolkit for modeling complex, nonlinear, and temporally dynamic relationships that characterize both macroeconomic systems and public health targets [6–8].

A substantial literature demonstrates the value of ML methods in economic forecasting. Ensemble models such as random forests and gradient boosting have outperformed traditional linear approaches in applications including GDP nowcasting, inflation forecasting, and macroeconomic risk assessment [9–11], in part because they capture nonlinear interactions and perform automatic variable selection in high-dimensional settings [12].

Related work has extended ML to health-relevant economic and environmental contexts. Satellite imagery has been used to estimate economic activity linked to healthcare access [13], computer vision methods have quantified built environments associated with neighborhood health disparities [14], and deep learning applied to central-bank communications has revealed latent economic signals predictive of market behavior [15]. In public health forecasting, models such as LSTM networks and gradient boosting have improved disease outbreak prediction and hospital resource planning, with reported accuracies exceeding 88% in some settings [16].

Despite growing recognition of the interdependence between economic conditions and health system capacity—highlighted during shocks such as the COVID-19 pandemic [17]—the integration of structured macroeconomic indicators into public health forecasting remains limited. ML models in this area are increasingly used not as ends in themselves, but as flexible tools for extracting signal from complex data. Prior work has shown that stochastic-gradient–based optimization with regularization and learning-rate control can achieve stable convergence in volatile economic time series [18], while interpretable ensemble and semi-parametric models have supported policy-relevant inference [19,20].

In parallel, causal machine learning frameworks—such as causal forests and generalized random forests—have expanded the role of ML in policy analysis [21,22]. Although the present study does not seek to estimate causal effects, it aligns with this broader objective by using predictive modeling to inform health-aligned decision-making under uncertainty.

Finally, much of the existing ML literature in public health focuses on clinical prediction or unstructured social determinants (e.g., electronic health records, text, or mobility data). By contrast, structured macroeconomic panel data—while central to fiscal and labor policy—have received comparatively little attention as predictive inputs for public health targets, despite evidence linking economic contractions to healthcare disruptions [2,23,24].

### Study objectives and contribution

The goal of this study is not to identify a single optimal predictive model, but to assess how informative macroeconomic indicators are for forecasting public health targets across targets and modeling paradigms [25]. Accordingly, we consider a variety of ML and statistical models to assess how a range of macroeconomic indicators can predict the public health targets [22]. The list of models includes optimization-based neural networks, generalized additive models, time-series models, and ensemble methods—treating these models as complementary analytical lenses. Recent reviews (e.g., Kasula et al. [26]) highlight the growing use of probabilistic and machine learning approaches across domains; however, their application to population-level public health forecasting using macroeconomic time-series remains limited. Our primary evaluation is based on a fixed training–testing split, with additional rolling-window strategies used to assess robustness under alternative forecasting scenarios. By comparing error profiles across targets and models, we aim to identify which public health targets exhibit a stable macroeconomic signal. This work contributes to the digital public health literature by systematically evaluating macroeconomic indicators for population health forecasting and assessing robustness across diverse modeling approaches and evaluation mechanisms [27,28]. To assess the informative value of macroeconomic indicators, we employ a comparative modeling framework that includes both baseline time-series models and models incorporating macroeconomic predictors. This design allows us to examine whether macroeconomic indicators provide consistent predictive signal beyond inherent temporal dynamics.

## Materials and methods

### Data description

#### Public health targets.

We examined three targets designed to capture system-level dimensions of public health capacity and access in the United States. These targets were obtained at the monthly level, seasonally adjusted, from the Federal Reserve Bank of St. Louis’s Federal Reserve Economic Data (FRED [29]) platform, which disseminates series produced by U.S. federal statistical agencies, including the U.S. Bureau of Labor Statistics (Current Employment Statistics) and the U.S. Census Bureau. Together, these public health targets represent complementary dimensions of public health system resilience: labor, organizational entry, and physical infrastructure. To ensure a balanced dataset where all targets and features are fully available, we used a study period spanning February 2005 through February 2025. Although the balanced dataset is available from July 2004 through May 2025, we begin the usable forecasting sample in February 2005 and end in February 2025 to keep the fixed-split and rolling-window evaluation periods aligned to consistent calendar boundaries for comparability across models.

- **Health Care and Social Assistance Employees (EM.T) [30]**: This variable reflects the operational labor capacity of the U.S. healthcare system. Workforce size is a direct determinant of health service accessibility, provider-to-patient ratios, and surge capacity—especially during public health emergencies. Fluctuations in employment within this sector have been linked to care delays, burnout, and systemic bottlenecks in both urban and rural settings [31]. Recent evidence further highlights the critical role of workforce availability, with reductions in staffed hospital capacity during the COVID-19 pandemic contributing to projected hospital occupancy rates approaching critical thresholds in the coming decades [32].
- **Health Care and Social Assistance Business Applications (BA.T) [33]**: This indicator captures entrepreneurial activity and new market entries within the healthcare and social assistance sector. It reflects the formation of new clinics, support service providers, and specialty practices, all of which contribute to the system’s flexibility, innovation, and responsiveness to unmet needs. A rise in business applications often precedes greater service availability and diversity, especially in underserved communities [34]. Empirical evidence also suggests that expansion in retail healthcare providers is associated with increased utilization of alternative care settings and reduced reliance on emergency departments for low-acuity conditions [35].
- **Total Health Care Construction Spending (CTS.T) [36]**: Construction spending in the healthcare sector is a key forward-looking indicator of physical infrastructure capacity. It reflects investments in new hospitals, clinics, outpatient centers, and long-term care facilities—facilities critical for expanding care access, reducing geographic disparities, and accommodating future demand. Higher spending often precedes increases in service delivery capability [37]. Historical evidence further demonstrates that large-scale investments in healthcare infrastructure can substantially expand system capacity, with public funding initiatives leading to long-term increases in hospital bed availability [38].

**Fig 1:**
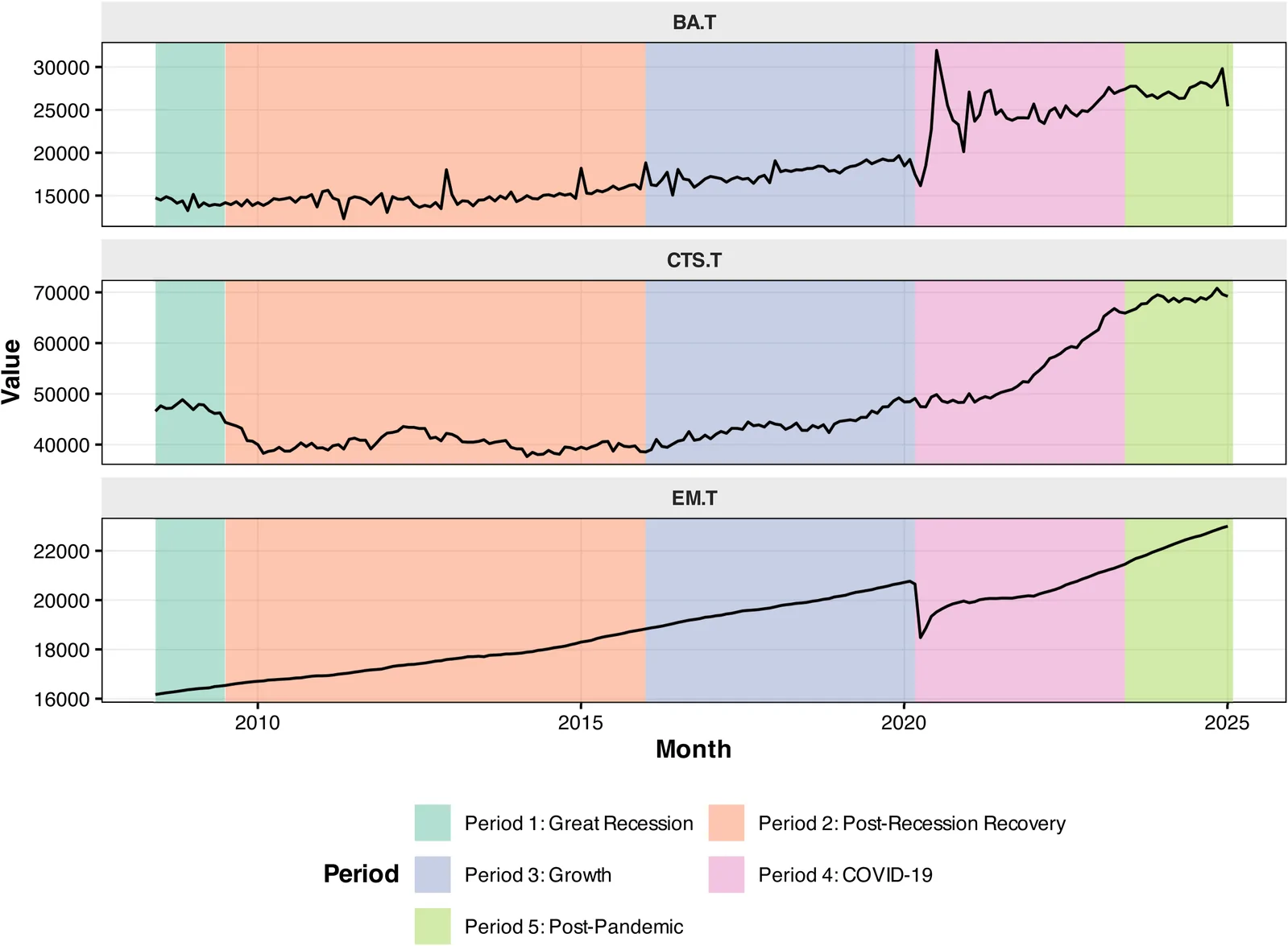
Temporal change of the targets over time periods.

To assess temporal variation in these public health targets, we evaluated their behavior across five U.S. socioeconomic periods. Fig 1 depicts their evolution across these periods, defined as follows:

- **Period 1 (June 2008 – June 2009):** The Great Recession
- **Period 2 (July 2009 – December 2015):** Post-Recession Economic Recovery
- **Period 3 (January 2016 – February 2020):** Pre-Pandemic Economic Growth
- **Period 4 (March 2020 – May 2023):** The COVID-19 Pandemic
- **Period 5 (June 2023 – January 2025):** Post-Pandemic Recovery

From Fig 1, we observed that EM.T exhibits a steady upward trajectory from Period 1 through the pre-pandemic period, with a noticeable but temporary decline at the onset of Period 4 (COVID-19), followed by a rapid recovery and continued growth. By contrast, CTS.T declines sharply during Period 1 (Great Recession), then stabilizes with moderate cyclical fluctuations through Periods 2 and 3, before accelerating markedly beginning in the latter half of Period 4. BA.T shows a gradual upward trend through Periods 2 and 3, experiences a brief disruption at the start of Period 4, and then rises sharply during the remainder of the pandemic and into the post-pandemic recovery period.

#### Macroeconomic indicators.

The U.S. Census measures 15 indicators of macroeconomic activity, collected from monthly and quarterly economic indicator surveys, available at the Census’s Economic Briefing Room. For each outcome, we use six macroeconomic indicators obtained at the monthly level, seasonally adjusted. Each corresponds to one of six core categories tracked by the US Census Bureau Economic Briefing Room: Business Profits & Formation, Inventories, Construction & Housing, Consumer Spending, Manufacturing, and International Trade [39], each relating to a category of economic activity (Business Profits & Formation, Inventories, Construction & Housing, Consumer Spending, Manufacturing, and International Trade).

These indicators reflect distinct but interrelated domains of economic activity that plausibly affect public health systems through labor markets, supply chains, consumer demand, and investment patterns. These indicators have been previously linked to upstream determinants of health system capacity and access [13,23].

- **Business Applications (BA.F) [40]**: Business applications serve as a leading indicator of economic expansion and job creation by capturing entrepreneurial activity and new business formation [34]. Empirical evidence further shows that business applications are strongly correlated with aggregate economic activity, reinforcing their role as a leading indicator [41].
- **Manufacturing and Trade Inventories (IN.F) [42]**: This indicator captures the combined business activities of retail trade, wholesale trade, and manufacturers, providing a broad measure of economic activity that encompasses production, business-to-business sales, and business-to-consumer transactions. Inventory levels in particular signal production adjustments and supply-demand imbalances, serving as an early warning system for economic slowdowns or expansions [43]. The series also feeds directly into estimates of gross domestic product and informs monetary and fiscal policy [43]. Prior work indicates that changes in inventory investment account for a substantial portion of production fluctuations during economic downturns, highlighting their importance in business cycle dynamics [44].
- **Total Construction Spending (CTS.F) [45]**: Total construction spending reflects long-term investment in infrastructure and built environment resilience, often preceding employment and service expansion [46]. Construction activity also constitutes a significant component of private investment and contributes materially to fluctuations in economic growth [47].
- **Retail and Food Services Sales (CNS.F) [48]**: Retail and food service sales are a key barometer of consumer sentiment and economic momentum, with direct implications for tax revenues and service demand [49]. Consumption expenditures account for a large share of gross domestic product, underscoring their central role in determining cyclical economic conditions [50].
- **Manufacturers’ Shipments, Inventories, and Orders (MN.F) [51]**: These indicators reflect production dynamics and industrial momentum. They are essential for assessing economic cycles and supply chain strength [52]. Manufacturing investment has been shown to explain a notable share of variation in economic output and downturns, particularly during major recessions [53].
- **Trade Balance in Goods and Services (IT.F) [54]**: The trade balance captures international demand, export competitiveness, and supply chain vulnerability—key factors in healthcare logistics and inflation sensitivity [55]. Fluctuations in trade balances are closely linked to exchange rate movements and price dynamics, with measurable pass-through effects on import prices and overall inflation [56,57].

**Fig 2:**
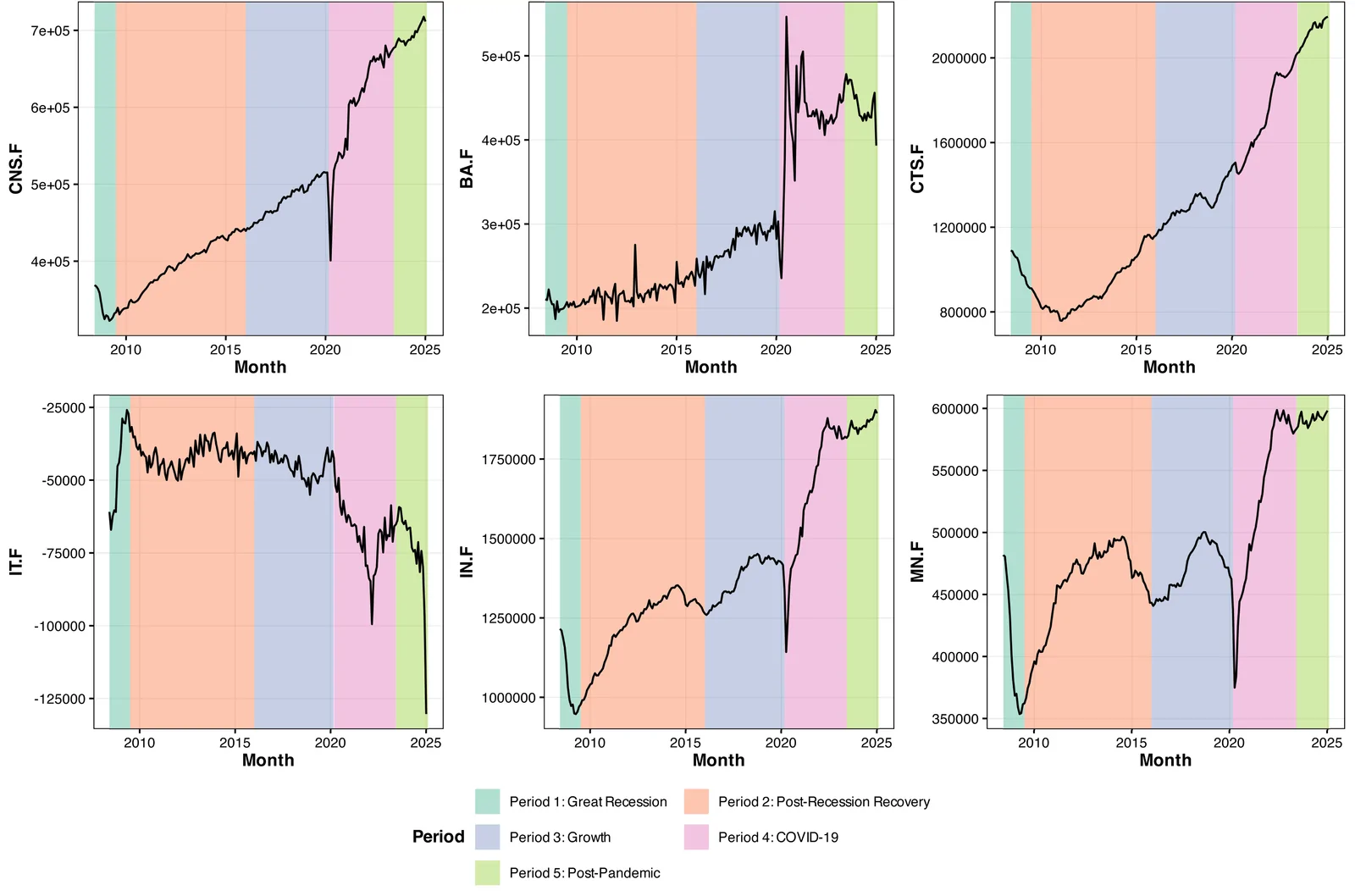
Monthly macroeconomic predictor features (BA.F, IN.F, CTS.F, CNS.F, MN.F, IT.F) over time; shaded regions denote the five socioeconomic periods.

Similar to the targets, we have also observed the temporal change of these macroeconomic indicators through Fig 2. Across the macroeconomic indicators, clear structural shifts are visible across recession, recovery, and pandemic periods. CNS.F shows a steady long-term upward trend from the beginning, with moderate cyclical fluctuations and a sharp but temporary decline at the onset of Period 4 (COVID-19), followed by rapid recovery and continued growth into Period 5. BA.F remains relatively stable through Periods 2 and 3, then exhibits a pronounced spike during the early COVID-19 period, consistent with pandemic-era business restructuring and rapid entry dynamics, before stabilizing at a higher post-pandemic level. CTS.F follows a cyclical investment pattern, declining during Period 1, recovering gradually through Periods 2 and 3, and accelerating sharply through the latter half of Period 4 and into Period 5, reflecting post-pandemic capital expansion. IT.F displays a downward trend through the pre-pandemic period, with pronounced volatility during Periods 3 and 4 and a sharp contraction during COVID-19, consistent with disruptions to global trade flows. IN.F demonstrates long-run growth with cyclical adjustments, interrupted by a brief contraction at the start of Period 4, followed by a steep post-pandemic expansion. Finally, MN.F exhibits repeated expansion–contraction cycles across Periods 2 and 3, a sharp decline at the onset of COVID-19, and strong rebound growth thereafter. Together, these trajectories illustrate coordinated macroeconomic shocks and recoveries that align temporally with major public health and economic crises, reinforcing the plausibility of these indicators as upstream macroeconomic signals of systemic health capacity. We did not apply dimensionality reduction (e.g., PCA), as the goal was to preserve the interpretability of macroeconomic indicators; models used are robust to correlated inputs.

Table 1 provides definitions of the public health targets and macroeconomic indicators used in this study for clarity.

**Table 1:** Definitions of public health targets and macroeconomic indicators.

Acronym	Full Name	Description
EM.T	Health Care and Social Assistance Employees	Monthly employment in the health care and social assistance sector, reflecting workforce capacity
BA.T	Health Care and Social Assistance Business Applications	New business applications in the health care and social assistance sector, indicating market entry and expansion
CTS.T	Total Health Care Construction Spending	Monthly spending on health care construction, reflecting infrastructure investment
BA.F	Business Applications	Total business applications across sectors, capturing entrepreneurial activity and economic expansion
IN.F	Manufacturing and Trade Inventories	Inventory levels indicating production and supply-demand dynamics
CTS.F	Total Construction Spending	Overall construction spending reflecting infrastructure investment trends
CNS.F	Retail and Food Services Sales	Consumer spending indicator reflecting economic activity and demand
MN.F	Manufacturers’ Shipments, Inventories, and Orders	Measures industrial production and supply chain activity
IT.F	Trade Balance in Goods and Services	Reflects international trade dynamics and economic conditions

Note: Acronyms ending with “T” are public health targets and acronyms ending with “F” are features or macroeconomic indicators.

All predictors and targets were obtained from publicly available government sources and reflect regularly released, seasonally adjusted series with relatively short publication lags (typically 1–2 months). Analyses were conducted using the most recent available data vintages, and predictors were lagged by one month to preserve temporal ordering. The detailed summary of the public health targets and the macroeconomic indicators has been tabulated in S1 Table of the supplementary material.

## Methodology

### Overview of modeling approaches

We evaluated multiple statistical and machine learning models to assess whether the predictive value of macroeconomic indicators for public health targets is consistent across different functional forms and learning paradigms [7,8,22,25]. Rather than seeking a single optimal model, this comparative design allows us to identify predictive patterns that persist across model classes. The approaches considered include optimization-based neural networks, generalized additive models, time-series models, and ensemble tree-based methods. Specifically, we consider:

- Optimization-based neural network regressors trained using stochastic gradient descent (SGD), Adaptive Moment Estimation (Adam), and Limited-memory Broyden–Fletcher–Goldfarb–Shanno (L-BFGS) optimizers [58,59];
- Generalized additive models (GAMs) to capture nonlinear but interpretable relationships [60,61];
- Autoregressive integrated moving average (ARIMA) models with exogenous regressors [62,63] to account for temporal dependence;
- Random forest regression models to capture nonlinear interactions and variable importance [10,64,65].

#### Modeling framework.

**Lagged prediction strategy:** All models are estimated using a common lag structure to ensure the temporal validity of the predictive relationship. For each outcome, macroeconomic indicators observed at month t−1 are used to predict the public health outcome measured at month *t* (or at the beginning of the corresponding forecas*t*ing window). We selected a one-month lag to reflect real-time data availability and to maintain a consistent modeling framework across approaches. Sensitivity analyses using alternative lag structures (e.g., 2–3 months) yielded similar results, with the one-month lag generally providing the best or comparable model fit. This alignment reflects a realistic temporal sequencing in which economic indicators are observed prior to downstream changes in public health targets, and it is consistent with operational forecasting workflows in which economic data become available before health outcomes are measured. All analyses were conducted on monthly time-series panel data consisting of three public health targets and six macroeconomic indicators. Each model uses macroeconomic indicators measured at month t−1 to predict the public health target at month *t*, ensuring temporal ordering of predictors and outcomes. For machine learning models, temporal dynamics are incorporated through the inclusion of a *t*ime index variable, allowing models to capture underlying trends alongside lagged macroeconomic predictors. It is to be noted that the models used here are for forecasting and association-based interpretation, not causal inference.

**Brief mathematical formulation:** Let $y_{t}$ denote a specific public health target at month *t* and let ${𝐗}_{t-1}\in {\mathbb{R}}^{p}$ denote the vector of macroeconomic indicators observed at month t−1, consistent with the lagged prediction strategy described above. Across all models, the objective is to learn a mapping


$$\begin{array}{c} y_{t}=f({𝐗}_{t-1};\;\theta ), \end{array}$$ (1)

where θ denotes the model parameters, and the prediction error is evaluated using squared or absolute deviations between $y_{t}$ and ${\hat{y}}_{t}$, where ${\hat{y}}_{t}$ is the prediction of $y_{t}$. Below, we provide a brief formulation of each model class; full derivations and implementation details are available in the GitHub repository: “public_health_forecasting_using_computationally_efficient_models” maintained under the username “shomechakraborty”.

**Neural network models (SGD, Adam, L–BFGS):** The neural network models assume a nonlinear prediction function f(·,θ) in equation 1, specified as a feed-forward neural network with parameters θ (weights and biases). Letting $h^{\left(0\right)}={𝐗}_{t-1}$, the network is defined recursively as


$$\begin{array}{c} h^{\left(\ell \right)}=\sigma^{\left(\ell \right)}(W^{\left(\ell \right)}h^{\left(\ell -1\right)}+b^{\left(\ell \right)}),\;\ell =1,\ldots ,L, \end{array}$$

where $\sigma^{\left(\ell \right)}(\cdot )$ denotes the activation function at layer ℓ, and the output is given by $f({𝐗}_{t-1};\theta )=h^{\left(L\right)}$. The parameters are estimated by minimizing the loss function


$$\begin{array}{c} ℒ(\theta )=ℒ(y_{t},f({𝐗}_{t-1};\;\theta )), \end{array}$$

where ℒ(θ) corresponds to either absolute error loss or squared error loss. Parameters θ are updated iteratively using a gradient-based rule


$$\begin{array}{c} \theta_{k+1}=\theta_{k}-\eta_{k}\;\nabla_{\theta }ℒ(\theta_{k}), \end{array}$$

where $\eta_{k}$ denotes the step size (learning rate). The three variants differ in how gradients and step sizes are computed:

- **SGD:** SGD is a simplified variant of gradient descent that updates parameters using randomly sampled data at each iteration. This stochastic approximation provides an efficient and scalable approach for large datasets, with simple updates and compatibility with momentum and learning-rate strategies, making it widely used in practice [66–68]. Under SGD, updates are computed using stochastic (mini-batch) gradients:
  $$\begin{array}{c} \theta_{k+1}=\theta_{k}-\eta_{k}\nabla {ℒ}_{B}(\theta_{k}), \end{array}$$
  where ${ℒ}_{B}$ denotes the mini-batch loss. A leaky ReLU activation function is used in hidden layers,
  $$\begin{array}{c} A_{j}^{\left(\ell \right)}=\left\{ \begin{array}{cc} S_{j}^{\left(\ell \right)}, & S_{j}^{\left(\ell \right)}>0,\\ \alpha S_{j}^{\left(\ell \right)}, & S_{j}^{\left(\ell \right)}\leq 0, \end{array}\right. \end{array}$$
  where $S_{j}^{\left(\ell \right)}$ is the pre-activation input and α>0 is a small constant.
- **Adam:** Gradients are adapted using first- and second-moment estimates [58],
  $$\begin{array}{c} m_{k}=\beta_{1}m_{k-1}+(1-\beta_{1})\nabla {ℒ}_{B}(\theta_{k}),\;v_{k}=\beta_{2}v_{k-1}+(1-\beta_{2})\nabla {ℒ}_{B}(\theta_{k}{)}^{2}, \end{array}$$
  with updates of the form
  $$\begin{array}{c} \theta_{k+1}=\theta_{k}-\eta_{k}\frac{{\hat{m}}_{k}}{\sqrt{{\hat{v}}_{k}}+\epsilon }. \end{array}$$
- **L–BFGS:** Updates follow a quasi-Newton direction,
  $$\begin{array}{c} \theta_{k+1}=\theta_{k}-\alpha_{k}H_{k}\nabla ℒ(\theta_{k}), \end{array}$$
  where $H_{k}$ is a low-rank approximation to the inverse Hessian matrix.

For Adam and L–BFGS models, hidden layers use the standard ReLU activation function


$$\begin{array}{c} A_{j}^{\left(\ell \right)}=\left\{ \begin{array}{cc} S_{j}^{\left(\ell \right)}, & S_{j}^{\left(\ell \right)}>0,\\ 0, & S_{j}^{\left(\ell \right)}\leq 0. \end{array}\right. \end{array}$$

All models employ a feed-forward architecture with three layers (input–hidden–output) consisting of 27×9×3 neurons. To ensure a fair and easily reproducible comparison, all baseline models were implemented using their standard framework settings. This is consistent with standard benchmarking practices in the literature, which recommend evaluating optimizers in their canonical form before extensive tuning to isolate architectural contributions from optimization artifacts [7,69].

**GAM:** The GAM specifies a semiparametric additive structure,


$$\begin{array}{c} y_{t}=\beta_{0}+\sum_{j=1}^{p}s_{j}(X_{t-1,j})+\varepsilon_{t},\;\varepsilon_{t}\sim \text{i.i.d. }(0,\sigma^{2}), \end{array}$$

where $\beta_{0}$ is the intercept, ϵ corresponds to error, $s_{j}(\cdot )$ are smooth spline functions estimated via penalized likelihood to balance fit and smoothness.

**ARIMA with exogenous macroeconomic indicators:** The outcome is modeled as a combination of autoregressive dependence, moving-average error dynamics, and lagged macroeconomic predictors:


$$\begin{array}{c} y_{t}=\sum_{i=1}^{p}\phi_{i}\;y_{t-i}+\sum_{j=1}^{q}\psi_{j}\;\varepsilon_{t-j}+{𝐗}_{t-1}^{⊤}\gamma +\varepsilon_{t},\;\varepsilon_{t}\sim 𝒩(0,\sigma^{2}), \end{array}$$

where $\phi_{i}$ are autoregressive coefficients, $\psi_{j}$ are moving-average coefficients, and γ represents the effects of the lagged macroeconomic indicators ${𝐗}_{t-1}$. The ARIMA components (including differencing and autoregressive/moving-average orders) were selected using an automated information-criterion–based procedure (auto.arima) to ensure consistent and data-driven model specification.

**Random Forest:** The Random Forest estimator forms an ensemble prediction


$$\begin{array}{c} y_{t}=\frac{1}{B}\sum_{b=1}^{B}f_{b}({𝐗}_{t-1}), \end{array}$$

where each $f_{b}(\cdot )$ is a regression tree trained on a bootstrap sample with random feature subsampling, allowing the ensemble to capture nonlinear effects and interactions while reducing variance.

Further details of the specified model are available on GitHub.

#### Evaluation design.

We evaluate model performance under two complementary forecasting designs. First, a fixed training–testing split (referred to as 80–20 hereafter), with approximately 80% of the series used for training the data. It trains models on data from February 2005 to January 2021 and evaluates them on the remaining 20% of the data, i.e., on February 2021–February 2025. Although the balanced dataset is available from July 2004, we begin the usable forecasting sample in February 2005 to accommodate the one-month lag structure (predictors at t−1 used to forecast outcomes at *t*) and to keep the evaluation periods aligned *t*o consistent calendar boundaries.

This setting reflects a standard forward-looking evaluation in which models are trained on historical observations and applied to later periods.

This fixed split serves as the primary evaluation framework, providing a stable setting for model estimation and comparison.

Second, a rolling 12–4 design trains on sequential 12-month windows and evaluates on the subsequent non-overlapping 4-month periods. This additional mechanism helps to examine temporal robustness beyond a single static split and to assess the sensitivity of the model performances. We also consider an additional alternative rolling horizons, such as a 6–1 scheme where models are trained on 6 consecutive months and evaluated on the subsequent 1-month test period.

These rolling-window schemes are used as complementary sensitivity analyses to assess short-term predictive performance under varying temporal contexts. The shorter training windows are not intended as primary evaluation frameworks, particularly for more flexible models that may require larger training samples, but rather to provide insight into temporal robustness. Rolling-window evaluation can also be informative in settings where recent observations are more relevant than distant history, such as during periods of structural change or evolving system dynamics, and it mimics operational forecasting scenarios in which models are updated as new data become available. This design also allows us to assess model robustness in the presence of structural changes and non-stationarity, including major economic disruptions such as the Great Recession and the COVID-19 pandemic.

The results from this mechanism are provided in S2 Table and S1 Fig of the Supplement.

#### Performance metrics.

The performance of each model for each design mechanism was summarized using mean absolute error (MAE), root mean squared error (RMSE), and mean absolute scaled error (MASE), which capture the average error magnitude, sensitivity to large deviations, and scale-adjusted error relative to a naive baseline, respectively. For observed public health targets $y_{t}$ and predictions ${\hat{y}}_{t}$ over a test set of size *n*,


$$\begin{array}{c} \text{MAE}=\frac{1}{n}\sum_{t=1}^{n}|y_{t}-{\hat{y}}_{t}|,\;\text{RMSE}=\sqrt{\frac{1}{n}\sum_{t=1}^{n}(y_{t}-{\hat{y}}_{t}{)}^{2}}. \end{array}$$

The 95% confidence intervals for MAE were obtained using a bootstrap procedure applied to the test-period prediction errors, which provides an empirical estimate of uncertainty and partially accounts for temporal dependence in the error structure.

We additionally report the mean absolute scaled error (MASE), defined as


$$\begin{array}{c} \text{MASE}=\frac{\frac{1}{n}\sum_{t=1}^{n}|y_{t}-{\hat{y}}_{t}|}{\frac{1}{n-1}\sum_{t=2}^{n}|y_{t}-y_{t-1}|}, \end{array}$$

which scales the forecast error relative to a one-step naive forecast based on the previous observation. This metric provides a robust, scale-independent benchmark for comparing predictive performance across models and targets. MASE values greater than 1 indicate that a model underperforms the naive one-step baseline, and substantially elevated values reflect poor predictive performance for those configurations rather than computational errors.

We do not conduct formal statistical tests of differences in forecast accuracy (e.g., Diebold–Mariano tests). The short test windows in the rolling schemes render such tests severely underpowered, and pairwise comparisons across six models, three targets, and three evaluation schemes would require substantial multiple testing corrections that complicate interpretation. The primary objective is descriptive comparison of performance patterns across models and evaluation frameworks rather than establishing formal model superiority.

#### Model implementation.

All models were implemented using widely adopted statistical software to ensure transparency and reproducibility. Default parameter settings were used for baseline estimation, consistent with recommendations to avoid conflating performance differences with extensive hyperparameter tuning [69]. Sensitivity analyses using alternative hyperparameter configurations are reported in S3 Table of the Supplementary Material. The neural network models (SGD, L–BFGS, and Adam) were implemented in Python, whereas the GAM, ARIMA, and Random Forest models were implemented in R. All analysis scripts and supporting code will be made publicly available in a GitHub repository to facilitate replication and reuse.

## Results

### Evidence of predictive signal in macroeconomic indicators

To assess whether macroeconomic indicators contain predictive signal beyond the inherent autoregressive structure of the series, we compared baseline ARIMA models with the ARIMA models that include individual macroeconomic predictors under the 80–20 split. The objective of this analysis is not to identify an optimal subset of predictors, but to evaluate whether specific indicators provide additional information for forecasting.

Table 2 summarizes the model fit statistics for ARIMA models with individual predictors. The results indicate that the contribution of macroeconomic indicators is target-dependent. For example, for EM.T and BA.T, certain indicators (e.g., consumer spending and business application measures) improve model fit relative to the baseline ARIMA model. In contrast, for CTS.T, the baseline ARIMA model remains competitive, with limited improvement from the inclusion of individual predictors.

**Table 2:** AIC, BIC for ARIMA models with individual predictors.

Target	Predictors	AIC	BIC
EM.T	CNS.F	2415.02	2434.50
	IN.F	2467.06	2480.05
	MN.F	2474.85	2491.09
	Intercept	2481.39	2497.62
	BA.F	2481.74	2491.48
	IT.F	2483.35	2502.83
	CTS.F	2573.12	2582.85
BA.T	BA.F	3261.01	3273.99
	IN.F	3263.03	3285.76
	MN.F	3268.29	3294.27
	IT.F	3269.06	3285.30
	CNS.F	3271.69	3294.45
	Intercept	3272.57	3285.56
	CTS.F	3273.49	3289.72
CTS.T	Intercept	3097.74	3107.48
	IT.F	3099.50	3112.48
	MN.F	3099.69	3112.68
	IN.F	3115.22	3144.49
	BA.F	3116.34	3145.61
	CNS.F	3118.40	3134.66
	CTS.F	3122.18	3138.44

Overall, these findings suggest that macroeconomic indicators can provide meaningful predictive information for some public health targets, but their contribution is selective rather than uniform across outcomes.

### Overall predictive performance

Across the primary 80–20 evaluation, the predictive value of macroeconomic indicators varied across public health targets (Table 3). Errors were lowest for EM.T across most models, indicating that macroeconomic indicators captured a larger share of variation for this target compared with BA.T and CTS.T. By contrast, BA.T exhibited moderately higher error magnitudes, while CTS.T generally showed the largest errors across models, suggesting that this target was more difficult to predict from the available macroeconomic inputs.

**Table 3:** Overall predictive performance across models and targets under the 80–20 and 12–4 evaluation schemes.

Targets	Models	80 – 20			12 – 4		
		MAE (95% CI)	RMSE	MASE	MAE (95% CI)	RMSE	MASE
**EM.T**	SGD	4459.52 (4328.05, 4590.98)	4572.99	67.59	431.40 (389.10, 473.71)	539.20	7.70
	Adam	16242.77 (15835.55, 16649.99)	16542.75	246.20	12030.10 (11010.16, 13050.04)	14336.72	214.82
	L-BFGS	3775.77 (3570.46, 3981.09)	4093.48	57.23	478.27 (373.44, 583.10)	933.38	8.54
	GAM	1469.19 (1136.64, 1811.67)	1932.44	28.05	379.43 (322.37, 445.60)	627.64	6.75
	ARIMA	722.63 (622.67, 819.71)	809.41	13.80	119.05 (78.41, 166.42)	371.40	2.10
	Random Forest	1321.69 (1050.20, 1598.47)	1664.03	25.24	206.67 (183.50, 235.19)	284.71	3.68
**BA.T**	SGD	4231.46 (3888.93, 4573.98)	4986.33	5.43	1552.79 (1245.18, 1860.40)	2818.38	1.87
	Adam	11186.67 (10764.19, 11609.16)	11650.22	14.35	12968.64 (12065.08, 13872.20)	14694.10	15.61
	L-BFGS	2376.28 (2111.66, 2640.90)	3130.45	3.05	1237.11 (1005.69, 1468.54)	2159.09	1.49
	GAM	3819.55 (3419.42, 4245.53)	4113.80	4.71	3118.54 (2595.21, 3698.29)	5323.15	3.79
	ARIMA	1547.64 (1332.18, 1763.15)	1736.88	1.91	1712.03 (1430.00, 2010.80)	2815.68	2.05
	Random Forest	1950.86 (1582.98, 2337.85)	2403.03	2.41	876.66 (711.41, 1069.55)	1695.44	1.07
**CTS.T**	SGD	18905.24 (17632.50, 20177.99)	21295.07	26.75	2742.23 (2447.00, 3037.45)	3551.82	4.14
	Adam	4184.56 (3867.89, 4501.23)	4843.33	5.92	2050.68 (1838.15, 2263.21)	2616.49	3.10
	L-BFGS	3140.23 (2824.58, 3455.89)	3971.19	4.44	1539.46 (1321.56, 1757.37)	2268.47	2.33
	GAM	8345.71 (6750.99, 10000.09)	10156.23	12.81	2362.33 (2085.58, 2659.07)	3224.32	3.61
	ARIMA	8055.80 (6718.66, 9435.21)	9447.69	12.36	1864.03 (1637.44, 2113.60)	2637.73	2.85
	Random Forest	13163.08 (11107.36, 15248.74)	15133.57	20.20	1362.92 (1207.62, 1528.66)	1816.46	2.09

Consistent with this pattern, under the 80–20 split the ARIMA achieved an RMSE of 809.41 for EM.T, compared with an RMSE of 1736.88 for BA.T. For CTS.T, error magnitudes were substantially larger across models, reflecting weaker alignment between the macroeconomic indicators and this target under the present modeling framework.

**Fig 3:**
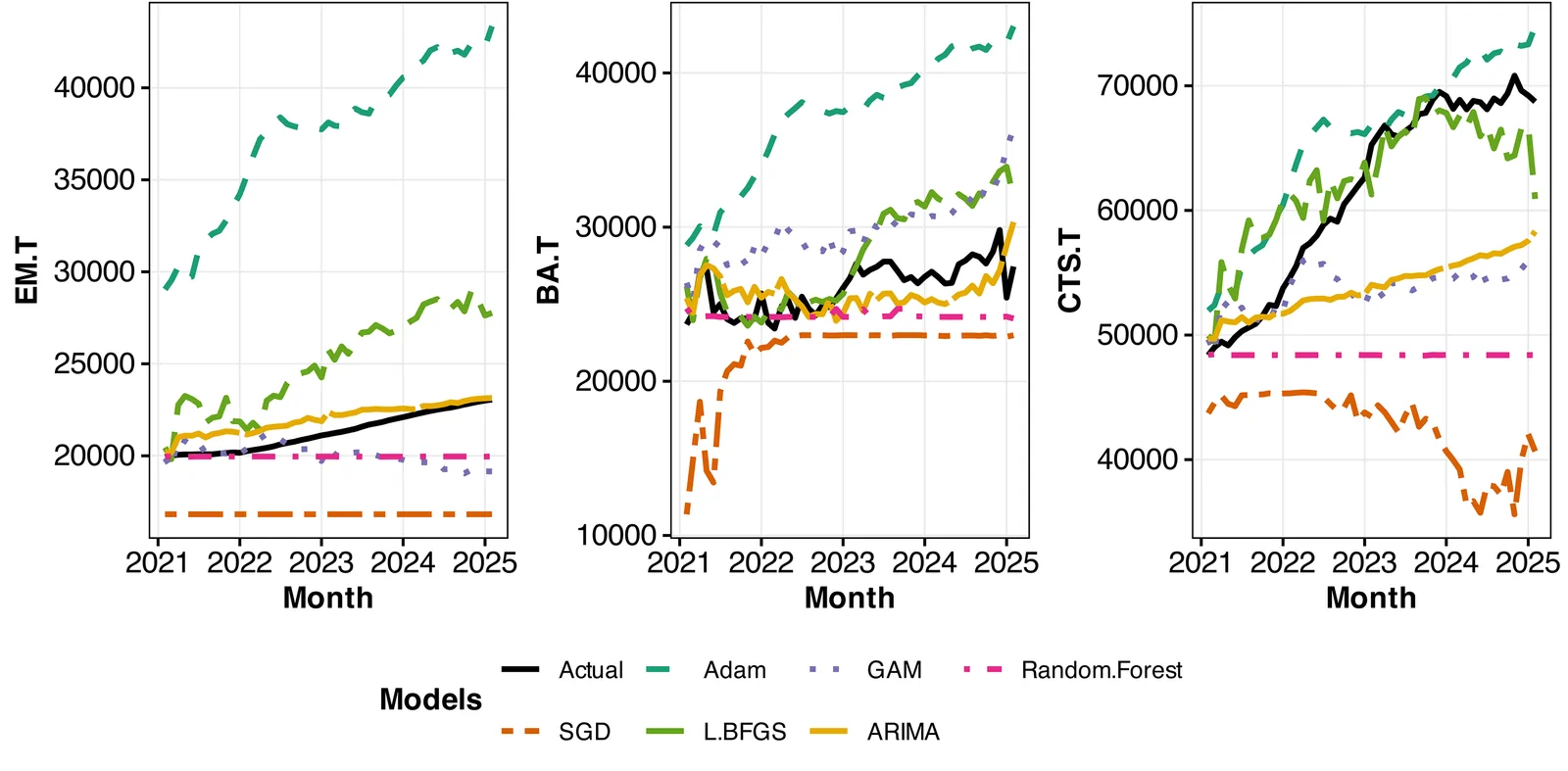
Comparing predicted targets from different models under the 80–20 mechanism.

No single model uniformly dominated across targets. Rather, performance tended to vary by target (Table 3), indicating that differences in predictive accuracy were driven more by the underlying predictability of each target than by algorithm choice alone. This pattern is also reflected in Fig 3, where predicted trajectories for EM.T generally track the observed series more closely than those for BA.T or CTS.T, and greater dispersion across models is visible for the less predictable targets.

Overall, under the 80–20 evaluation, Random Forest and ARIMA were among the most consistent performers for EM.T and BA.T based on RMSE and MASE values, followed by GAM and L–BFGS. For CTS.T under the same scheme, the strongest performance was observed for L–BFGS and Adam, followed by ARIMA, while GAM and Random Forest showed higher overall error levels. Under the 12–4 rolling evaluation, the broad ranking of models remained similar, with Random Forest, ARIMA, and L–BFGS generally among the top performers. Results from the 6–1 scheme (reported in the Supplement) showed a similar pattern, although the performance of GAM and ARIMA tended to degrade as the training window shortened, likely reflecting the reduced amount of information available for model fitting.

### Temporal robustness and prediction trajectories

**Fig 4:**
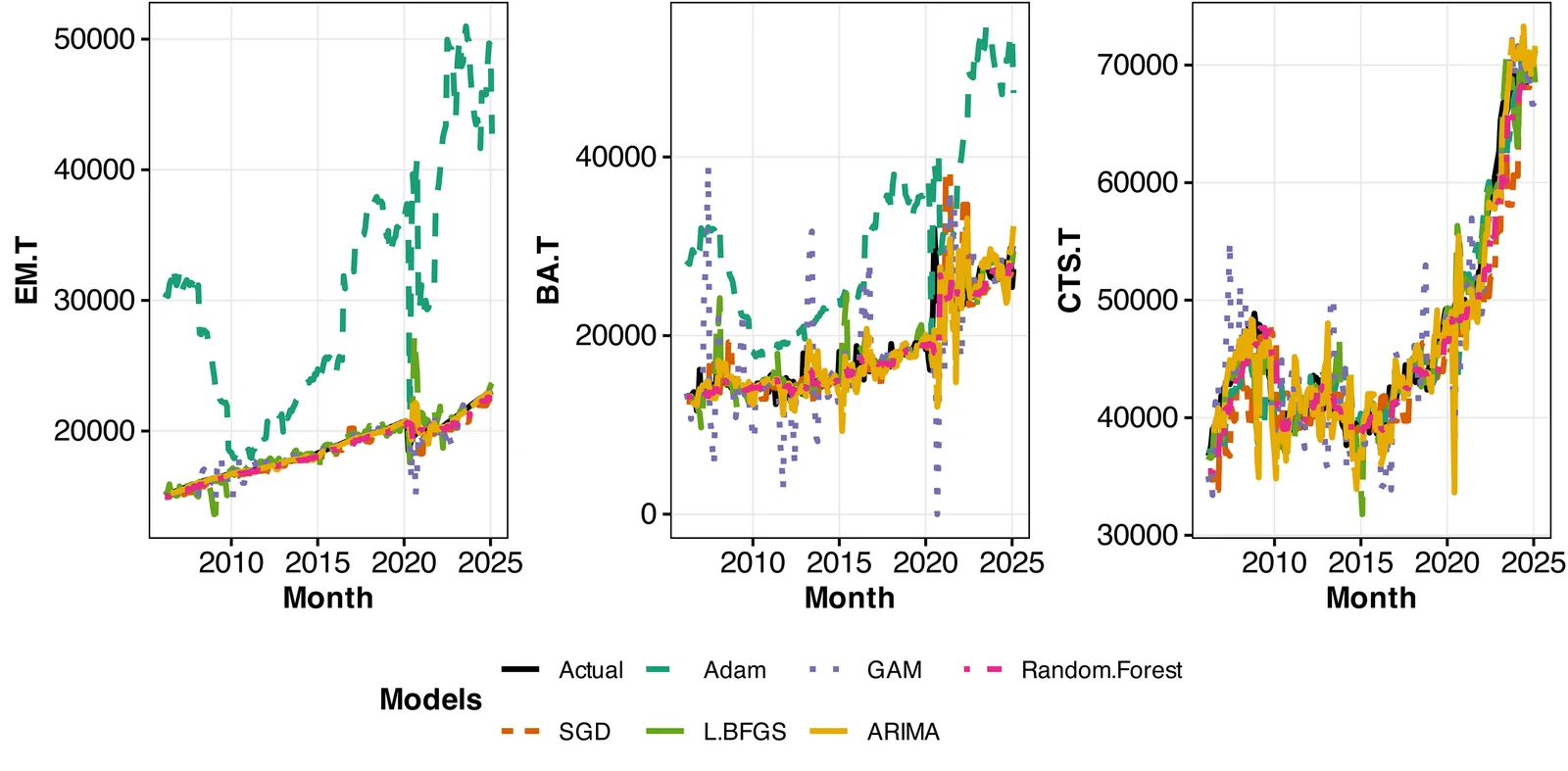
Comparing predicted targets from different models under the 12–4 mechanism.

Findings were broadly consistent under the rolling 12–4 evaluation (Fig 4). Public health targets that showed strong macroeconomic signal in the 80–20 analysis (e.g., EM.T) continued to produce stable prediction accuracy across rolling windows, whereas BA.T displayed greater temporal variability and smoother but less responsive prediction trajectories. As expected, absolute error levels increased modestly due to shorter training horizons, but the relative ordering of target difficulty and model performance remained similar to the primary evaluation.

Taken together, these results indicate that macroeconomic indicators contain meaningful predictive information for certain health system targets—particularly those related to workforce capacity—while predictive value is more limited for others. The stability of findings across models and evaluation strategies supports the interpretation that patterns reflect underlying macroeconomic signals rather than model-specific artifacts. These findings support the interpretation that observed predictive performance (Fig 3) reflects intrinsic signal strength rather than overfitting or algorithmic artifacts. Results are interpreted as descriptive comparisons of predictive performance across models and evaluation designs, rather than formal statistical comparisons.

In addition, visual inspection of Figs 3 and 4 suggests that several models produce smoother trajectories than the observed series and may not fully capture abrupt level changes or periods of rapid trend acceleration. This pattern indicates limited responsiveness to short-term fluctuations, consistent with underfitting or lagged adjustment rather than effective noise reduction.

### Results from other mechanism

Under the 6–1 rolling evaluation scheme—where models are trained on six months of data and tested on the subsequent month—performance patterns were broadly consistent with those observed in the primary analyses. As summarized in S2 Table and illustrated in S1 and S2 Figs, the Random Forest model demonstrated the strongest overall performance across targets, yielding the lowest MAE and RMSE values for EM.T, BA.T, and CTS.T (e.g., EM.T MAE = 112.18). The L-BFGS neural model also performed competitively for CTS.T and EM.T, whereas Adam and GAM consistently exhibited poorer accuracy, with substantially higher errors across public health targets. These 6–1 results, included in the Supplementary Materials, reinforce the robustness of the core findings across alternative temporal evaluation frameworks.

## Discussion

Our results indicate that some public health targets exhibit stronger and more stable predictive alignment with macroeconomic indicators than others, particularly under forecasting-oriented evaluation designs. These relationships are most evident for targets linked to structural system conditions such as labor capacity and market activity. The findings should be interpreted as evidence of predictive associations rather than causal effects. The models evaluate whether macroeconomic indicators contain signals useful for forecasting public health targets, rather than identifying economic mechanisms or intervention effects. Across both evaluation schemes (the 80–20 fixed split and the rolling 12–4 framework), models demonstrated consistent directional alignment between macroeconomic features and health system targets, while also revealing periods in which performance differed in response to volatility and structural regime shifts. These results reinforce evidence that economic fluctuations propagate through workforce, capital, and service-delivery channels in ways that shape downstream public health access and resilience [4,10,18,70,71]. Observed differences in model performance should be interpreted cautiously, as they may reflect model specification, tuning choices, training window length, data-regime dependence, and other data characteristics rather than intrinsic superiority of any specific modeling approach.

A central contribution of this work is the comparative evaluation of forecasting models in a health-relevant macroeconomic context. More flexible learning architectures (such as Adam-optimized neural networks and GAM-based models) were able to capture nonlinear temporal variation, but in some settings their performance degraded during shock and transition periods, particularly around the COVID-19 disruption. This observation is consistent with prior work suggesting that aggressively adaptive optimization strategies may be less stable in nonstationary environments [72,73]. By contrast, models with stronger implicit regularization and smoother optimization dynamics, including SGD-based and L–BFGS neural models, demonstrated more stable error behavior and closer alignment with observed outcome trajectories across targets, echoing prior findings on the generalization advantages of constrained or structure-aware training in economic time-series modeling.

These differences are not only methodological but also practically relevant for health-system planning. Forecasting tools intended to support preparedness and resource allocation must balance predictive accuracy with reliability, computational tractability, and transparency—particularly in settings with limited analytic capacity. Models that perform consistently across economic regimes are better positioned to inform anticipatory decisions such as workforce stabilization, infrastructure investment timing, and surge-capacity planning, aligning with broader priorities in digital health regarding deployability and equity-sensitive decision support.

Several design choices introduce important limitations. First, the analysis uses a one-month lag structure; alternative lag horizons or distributed-lag specifications may reveal different temporal relationships. Second, the set of macroeconomic indicators and public health targets is necessarily selective, and different indicator combinations may yield different forecasting values. Third, model evaluation focuses on aggregate predictive accuracy rather than feature-specific attribution, and future work should examine which indicators contribute most strongly to forecast performance across periods. Fourth, the findings are strictly based on the economy and public health infrastructure within the U.S. and, hence, may not generalize to other countries or health-system contexts. Finally, the performance of classical statistical models was sensitive to the evaluation design. Under the rolling 12–4 scheme, ARIMA produced missing predictions in early test windows due to standard initialization constraints when fitting ARIMA models on short training sequences, an issue not observed under the fixed 80–20 split, where longer historical series were available. GAM performance similarly degraded under shorter training horizons (e.g., the 6–1 scheme), reflecting limited information for stable smooth estimation. Together, these patterns highlight the greater robustness of the machine learning models across varying training–testing configurations. Future work should explore alternative lag structures, cross-context generalization, and deployment-focused evaluation to assess whether such forecasting pipelines can be integrated into operational public-health monitoring environments. An additional limitation is that the analysis is based on revised data and does not explicitly account for real-time data availability or subsequent data revisions, which may influence forecasting performance in operational settings.

From a digital-health perspective, these results suggest that routinely collected macroeconomic indicators may serve as an upstream data stream for monitoring structural stress in health-system capacity [74]. Rather than replacing clinical surveillance, such forecasts may complement operational planning by identifying periods when capacity trends begin to diverge from historical patterns.

## Conclusion

This study shows that macroeconomic indicators can provide useful lead-time signals about structural conditions in the U.S. health system and that these signals can be translated into meaningful forecasts using computationally efficient and statistically stable modeling approaches. Across two complementary evaluation frameworks and multiple model classes, we observed consistent predictive alignment between economic precursors and health-system targets, with models emphasizing stability and implicit regularization performing more reliably during periods of volatility.

The contribution of this work lies not only in model comparison but in framing macroeconomic forecasting as a component of digital public health infrastructure. The results suggest that economic surveillance can complement clinical and epidemiologic monitoring by supporting anticipatory awareness of system-level pressures relevant to planning, preparedness, and policy response. At the same time, the findings should be interpreted in light of key constraints, including the reliance on a single-lag specification, a selected set of macroeconomic indicators and capacity-focused outcome measures, and the absence of causal identification or feature-level attribution. These factors highlight the need for cautious interpretation and motivate extensions that explore broader indicator sets, alternative lag structures, and outcome domains.

Future research should build on this work by integrating macroeconomic signals with other data streams, examining feature- and mechanism-specific pathways, and evaluating transportability across institutional and geographic contexts. Such efforts will help advance economic-to-health forecasting as a practical, equity-aware digital health capability that supports more anticipatory and resilient health-system governance.

## Supporting information

S1 Fig Comparing predicted targets from different models under the 6–1 mechanism. GAM and Adam show substantially larger deviations from observed values compared to other models, while SGD, L-BFGS, and Random Forest track the observed series more closely across all three targets.
(EPS)

S2 Fig Comparing predicted targets from best performing models under the 6–1 mechanism. Random Forest, SGD, and L-BFGS demonstrate the closest alignment with observed trajectories across EM.T, BA.T, and CTS.T under the 6–1 scheme.
(EPS)

S1 Table Summaries of targets and features across periods. This table provides the minimum, maximum, mean, and standard deviation of each public health target and macroeconomic indicator across the five socioeconomic periods from February 2005 to February 2025.
(XLSX)

S2 Table Overall predictive performance across models and targets under the 6–1 evaluation scheme. This table reports MAE (with 95% confidence intervals), RMSE, and MASE for all models and targets under the 6–1 rolling window mechanism, with Random Forest achieving the lowest errors across all three targets.
(XLSX)

S3 Table Model performance under alternative hyperparameter configurations. This table reports MAE, RMSE, and MASE for SGD, Adam, and L-BFGS under three hyperparameter settings — standard (Regular HP), AHP1, and AHP2 — across all targets and evaluation schemes. SGD shows low variability across configurations, Adam exhibits substantial sensitivity with frequent performance degradation, and L-BFGS remains relatively stable under moderate changes but deteriorates under more extreme configurations. Overall conclusions regarding target-dependent predictability remain consistent across settings.
(XLSX)
