# Supplementary material for: Assessing the informative value of macroeconomic indicators for public health forecasting
Source: PLOS Digit Health. 2026 Aug 20;5(8):e0001599. doi: 10.1371/journal.pdig.0001599 (PMC13492747; doi:10.1371/journal.pdig.0001599)
Supplement: Supplementary file 1 [file pdig.0001599.s007.pdf]

Response to Reviewers

Manuscript ID: **PDIG-D-26-00132**

Title: *Assessing the Informative Value of Macroeconomic Indicators for Public Health Forecasting*

Journal: *PLOS Digital Health*

Dear Dr. Luo and Reviewers,

We sincerely thank the editor and reviewers for their careful evaluation of our manuscript and for the constructive suggestions that helped improve the clarity and rigor of the work. We have carefully revised the manuscript to address all comments. Below we provide a point-by-point response to the issues raised. Reviewer comments are summarized and grouped where multiple reviewers raised similar concerns. The corresponding revisions are indicated in the manuscript.

Summary of Major Revisions

The revision primarily addresses the following areas:

- Expanded methodological details, including neural network architecture, ARIMA specification, lag selection, and evaluation design.
- Improved clarity and framing in the Introduction, including definitions of public health targets and methodological overview.
- Added analyses to assess predictive signal (baseline vs. macroeconomic models) and hyperparameter sensitivity.
- Enhanced evaluation framework with additional metrics (MASE) and clarified uncertainty estimation (bootstrap-based CIs).
- Revised Results and Discussion to adopt more cautious, evidence-aligned interpretations and avoid overstated claims.
- Addressed concerns on non-stationarity, real-time data validity, and rolling-window evaluation.

Specific Responses to Reviewer Comments

1. “The authors should provide detailed information on the neural network architectures, including the number of layers and the activation functions used, as well as the. optimization strategies.”

Response: Thanks to the reviewer for suggesting this. We have added additional modeling details in the “Modeling Framework” subsection of the Methodology section.

2. “To support their claim that these indicators are "leading signals," the authors should conduct cross-correlation analysis or use AIC/BIC to find the most predictive time lags for each indicator.”

Response: We thank the reviewer for this suggestion. Our objective was not to optimize lag structure for each predictor, but to evaluate whether macroeconomic indicators provide consistent predictive signal under a common and operationally meaningful forecasting framework. We therefore adopted a fixed one-month lag to ensure temporal validity and comparability across models, reflecting real-time availability of macroeconomic indicators prior to public health outcomes.

To assess robustness, we conducted a sensitivity analysis using alternative lag specifications (e.g., 2–3 months). Across targets, the one-month lag consistently provided the best or comparable model fit based on information criteria (AIC/BIC). For example, under the GAM framework, the one-month lag yielded lower AIC/BIC compared to longer lags for BA.T, EM.T, and CTS.T. These findings support the use of a one-month lag, and we therefore retained this specification for all primary analyses. See the following Table R1 for complete AIC, BIC comparison.

Table R1: Table showing AIC and BIC values under GAM model for different lags. Smallest AIC and BIC per target has been highlighted

Target	Model	AIC			BIC		
		Lag = 1 month	Lag = 2 months	Lag = 3 months	Lag = 1 month	Lag = 2 months	Lag = 3 months
BA.T	GAM	3241.97	3266.78	3251.24	3284.34	3313.16	3301.96
CTS.T	GAM	3160.00	3182.54	3188.94	3211.88	3234.30	3240.52
EM.T	GAM	2569.43	2660.76	2697.20	2621.08	2712.31	2748.67

Based on the above, we have added the following text to the modeling framework to highlight our choice.

"We selected a one-month lag to reflect real-time data availability and to maintain a consistent modeling framework across approaches. Sensitivity analyses using alternative lag structures (e.g., 2–3 months) yielded similar results, with the one-month lag generally providing the best or comparable model fit."

3. "The authors need to explain how the models were recalibrated for "Period 4" (COVID-19), since this period likely disrupted previous weightings."

Response: We thank the reviewer for this comment. We did not explicitly recalibrate models for Period 4 (COVID-19). Instead, our evaluation framework inherently accounts for such structural changes through the training–testing design. In the fixed 80–20 split, models are trained on pre-pandemic data and evaluated on later periods, including COVID-19, thereby assessing their ability to generalize under structural disruption. In the rolling-window analyses, models are repeatedly re-estimated on temporally localized data, allowing parameters to adapt implicitly to changing regimes, including the pandemic period. Thus, rather than explicit recalibration, our approach evaluates model robustness to such structural shifts.

4. "In the Methodology section, specifically in the overview of modeling approaches. It is suggested to refer from the paper: " A Comprehensive Review of Artificial Intelligence Models for

Lifetime Value Optimization “. This reference provides a useful comparison for probabilistic and machine learning models. Since the manuscript looks at different modeling methods for long-term health system targets, this citation supports the choice of ensemble and deep learning method over deterministic ones.”

Response: Thank you for this helpful suggestion. We agree that A comprehensive review of artificial intelligence models for lifetime value optimization provides a useful overview of modern AI and machine learning approaches, including probabilistic and deep learning models.

However, our manuscript differs in both objective and scope. The cited work is a review paper in a marketing/optimization context (customer lifetime value), whereas our study focuses on empirical evaluation of forecasting models for population-level public health outcomes using macroeconomic time-series data. As such, our goal is not to provide a comprehensive survey of AI methods, but rather to compare the predictive utility of selected model classes (e.g., ARIMA, neural networks trained via SGD/Adam/L-BFGS) within this specific application domain.

That said, we agree that the reference can help contextualize the broader landscape of machine learning approaches. We have incorporated this reference in the Introduction to contextualize the broader landscape of machine learning and probabilistic models, while keeping the Methodology section focused on the specific models implemented in this study. Based on that, we have added the following line in the Introduction section:

“*Recent reviews (e.g., Kasula et al., [26]) highlight the growing use of probabilistic and machine learning approaches across domains; however, their application to population-level public health forecasting using macroeconomic time-series remains limited.*”

5. “The authors should clarify whether they used any dimensionality reduction methods, such as PCA, before fitting models to address correlated economic features.”

Response: We thank the reviewer for this suggestion. We did not apply dimensionality reduction methods such as PCA prior to model fitting. Our objective was to evaluate whether macroeconomic indicators, as a set, provide useful predictive information for public health targets, rather than to identify an optimal reduced set of features. Accordingly, we retained the original indicators to preserve interpretability and to assess their collective contribution across modeling approaches. We have clarified that in the Materials and Method section by adding:

“*We did not apply dimensionality reduction (e.g., PCA), as the goal was to preserve the interpretability of macroeconomic indicators; models used are robust to correlated inputs.*”

6. “The authors should include a table that defines each acronym, such as BA.F and IN.F, to help non-economist readers.”

Response: We thank the reviewer for this helpful suggestion. To improve clarity for a broader audience, we have added a summary table (**Table 1**) at the end of the Data Description section that defines all acronyms used for the public health targets and macroeconomic indicators, along with their full names and brief descriptions. This allows readers to quickly reference the terminology while preserving the detailed explanations already provided in the text.

7. “Introduction: The introduction provides a strong rationale for the study, supported by a comprehensive literature review and a clearly articulated research question. To enhance clarity, I encourage the authors to define the public health targets and capacity measures at the outset and to briefly justify their selection as proxies for health system strain.

From a methodological perspective, the introduction should show greater specificity regarding the assessment of informative value. In particular, it should clarify whether the analysis examines incremental predictive value beyond trend and seasonality, delineate the role of baseline time-series models, and specify the approach to forecast performance validation, such as a fixed split, a rolling window, or both. Providing these details is essential to support the paper's contribution and to ensure alignment between the introduction and the subsequennt analytical claims. Thank you.”

Response:

Early definition and justification of public health targets: We thank the reviewer for this suggestion. We have revised the Introduction to briefly define the public health targets at the outset and to clarify their role as proxies for key dimensions of health system capacity, including workforce, organizational activity, and infrastructure. Keeping these in mind, we have added the following in the Introduction:

“*In this study, we focus on three public health targets that reflect key dimensions of health system capacity: workforce (health care and social assistance employment), organizational activity (health care business applications), and infrastructure investment (health care construction spending). These measures serve as proxies for health system strain, capturing complementary aspects of labor availability, service expansion, and capital investment.*”

Clarification of methodological framework in Introduction: We have also revised the Introduction to provide greater methodological clarity. Specifically, we now clarify that the study evaluates the informative value of macroeconomic indicators through comparative modeling frameworks, including baseline time-series models and models incorporating macroeconomic predictors. We further specify that model performance is assessed using both fixed train–test splits and rolling-window validation schemes to ensure robustness. Hence, we have added the following at the end of the Introduction section:

“*To assess the informative value of macroeconomic indicators, we employ a comparative modeling framework that includes both baseline time-series models and models incorporating macroeconomic predictors. Model performance is evaluated using a primary fixed train–test split, supplemented by rolling-window validation schemes to assess temporal robustness. This design allows us to examine whether macroeconomic indicators provide consistent predictive signal beyond inherent temporal dynamics.*”

8. “Methods: The methods section is robust and reproducible; however, there are several aspects that I think require clarification. While the one-month lag is appropriate, the manuscript should explicitly discuss real-time data availability, publication lags, and revision histories for both predictors and targets. Employing revised or seasonally adjusted series without specifying the information available at each time point may artificially enhance perceived predictabilityy.

The figures show clear structural breaks at the Great Reccession and the COVID-19 pandemic, yet the manuscript does not explain how nonstationarity and regime change are handled. ARIMAX requires explicit justification of differencing and seasonal term selection; ML models can inadvertently capture trend without explicit time features or de-trending. The central claim, that macroeconomic indicators improve forecast accuracy, also requires a clean baseline comparison: each model should have a counterpart using only autoregressive terms, with economic indicators added incrementally. Without this, the analysis cannot isolate the contribution of macroeconomic variables from the series' inherent extrapolabilityy. The 80–20 data split is appropriate; however, the rolling schemes (12–4 and 6–1) are unconventional and likely underpowered, especially for neural networks and random forests. The manuscript should justify the use of short training windows and detail the methods employed to control overfitting. An expanding-window rolling-origin evaluation would provide a more defensible approach and better reflect operational practice. Regarding evaluation metrics, NRMSE normalized by the test mean is unstable under level shifts; MASE or skill scores relative to a seasonal-naive or autoregressive baseline would offer a more rigorous assessment.

Default hyperparameters are insufficiently justified unless their suitability for this specific problem is explicitly shown. Limited, pre-specified tuning within appropriate time-series resampling frameworks is preferable. At a minimum, sensitivity analyses should confirm that the conclusions are not dependent on arbitrary default setting.. These suggestions should improve the methds section. Thank you."

Response:

Data availability & real-time validity: We thank the reviewer for this important point. All predictors and targets used in this study are derived from publicly available, regularly released government data sources (e.g., FRED and U.S. Census indicators), with publication lags that are generally short (on the order of 1–2 months for most series). In our modeling framework, predictors are lagged by one month relative to the target, which aligns with the typical data availability timeline and helps preserve temporal validity.

While we acknowledge that these data may undergo minor revisions over time, our analysis uses the most recent available vintage of each series. Our objective is to assess whether macroeconomic indicators contain predictive signal under a consistent and operationally meaningful forecasting setup, rather than to replicate real-time nowcasting under historical data vintages. We have clarified this point in the manuscript:

"*All predictors and targets were obtained from publicly available government sources and reflect regularly released, seasonally adjusted series with relatively short publication lags (typically 1–2 months). Analyses were conducted using the most recent available data vintages, and predictors were lagged by one month to preserve temporal ordering.*"

Non-stationarity & structural breaks: We thank the reviewer for this comment. We acknowledge that the time series exhibit structural changes across major economic periods, including the Great Recession and the COVID-19 pandemic. Rather than explicitly modeling regime changes, our approach evaluates model performance across these periods through the training–testing design. In the fixed 80–20 split, models trained on earlier periods are evaluated on later periods that

include structural disruptions, while the rolling-window analyses involve repeated re-estimation on temporally localized data. This framework allows us to assess the robustness of different modeling approaches under non-stationarity and regime changes without imposing explicit structural assumptions. Hence, we have added the following in the Methods section:

"*This design also allows us to assess model robustness in the presence of structural changes and non-stationarity, including major economic disruptions such as the Great Recession and the COVID-19 pandemic.*"

<u>ARIMAX specification clarity</u>: We thank the reviewer for this comment. The ARIMA models with predictors were specified using an automated selection procedure (auto.arima), which determines the appropriate order of differencing and inclusion of autoregressive and moving-average terms based on information criteria. This approach is widely used to ensure a data-driven and consistent specification across targets without imposing restrictive assumptions. As our goal is comparative evaluation across modeling frameworks rather than detailed time-series model specification, we adopted this standard procedure for ARIMA implementation. We add the following where we describe the ARIMA model:

"*The ARIMA components (including differencing and autoregressive/moving-average orders) were selected using an automated information-criterion–based procedure (auto.arima) to ensure consistent and data-driven model specification.*"

<u>ML models & trend handling</u>: We thank the reviewer for this comment. In our modeling framework, temporal dynamics are incorporated through the inclusion of a time index variable, which allows machine learning models to capture underlying trends in the data. Additionally, lagged macroeconomic predictors provide temporal structure aligned with the forecasting setup. Our objective is not to explicitly de-trend the series, but to evaluate predictive performance under a consistent framework across models. This approach allows different modeling strategies to capture temporal patterns, including trend, in a data-driven manner. We have explained this in our Method section:

"*For machine learning models, temporal dynamics are incorporated through the inclusion of a time index variable, allowing models to capture underlying trends alongside lagged macroeconomic predictors.*"

<u>Baseline vs macro models (incremental value)</u>: We thank the reviewer for raising this important point. To isolate the contribution of macroeconomic indicators beyond the inherent autoregressive structure of the series, we used ARIMA as the baseline comparator. This choice is principled rather than one of convenience: ARIMA is the standard autoregressive benchmark in time-series forecasting, and information criteria (AIC/BIC) — the natural and well-established tools for assessing incremental predictor value — are well-defined within this framework. By contrast, AIC/BIC do not have direct equivalents for neural network models, making ARIMA the most transparent and methodologically consistent framework for this comparison. Extending the baseline comparison to all model classes would require different and potentially incompatible evaluation criteria across models, complicating interpretation rather than clarifying it.

Our objective was not to identify an optimal subset of predictors, but to evaluate whether macroeconomic indicators individually provide additional predictive signal beyond the autoregressive baseline. These analyses consistently indicate that the contribution is **target-dependent**. For example, for EM.T and BA.T, certain indicators (e.g., consumer spending and business formation measures) improved model fit relative to the baseline, whereas for CTS.T, the baseline ARIMA model remained competitive or optimal (see Table R2).

While combinations of predictors can further improve model fit in some cases, these improvements are not uniform across targets. Taken together, these findings support our primary conclusion that macroeconomic indicators provide meaningful predictive information for some public health targets, but their contribution is selective rather than universally beneficial.

Table R2: AIC, BIC for ARIMA models with individual predictors

Target	Predictors	AIC	BIC
EM.T	CNS.F	2415.02	2434.50
	IN.F	2467.06	2480.05
	MN.F	2474.85	2491.09
	Intercept	2481.39	2497.62
	BA.F	2481.74	2491.48
	IT.F	2483.35	2502.83
	CTS.F	2573.12	2582.85
BA.T	BA.F	3261.01	3273.99
	IN.F	3263.03	3285.76
	MN.F	3268.29	3294.27
	IT.F	3269.06	3285.30
	CNS.F	3271.69	3294.45
	Intercept	3272.57	3285.56
	CTS.F	3273.49	3289.72
CTS.T	Intercept	3097.74	3107.48
	IT.F	3099.50	3112.48
	MN.F	3099.69	3112.68
	IN.F	3115.22	3144.49
	BA.F	3116.34	3145.61
	CNS.F	3118.40	3134.66
	CTS.F	3122.18	3138.44

To address this, we have added a new subsection in the Results section (“Evidence of predictive signal in macroeconomic indicators”) that explicitly evaluates the incremental predictive value of individual macroeconomic indicators relative to baseline ARIMA models.

Rolling window design concerns: We thank the reviewer for this thoughtful comment. The rolling-window schemes (12–4 and 6–1) were included as complementary sensitivity analyses to assess

short-term predictive performance under limited data scenarios, rather than as primary evaluation frameworks. Our primary results are based on the 80–20 split, which provides a more stable estimation setting.

We acknowledge that shorter training windows may be less suitable for more flexible models such as neural networks and random forests. Accordingly, these rolling-window results are interpreted cautiously and are intended to provide additional insight into temporal robustness rather than to serve as the basis for primary conclusions. At the same time, rolling-window evaluation can be useful in settings where recent observations are more informative than distant history, such as during periods of structural change, and it mimics short-term operational forecasting scenarios where models are repeatedly updated using the most recent data. We have clarified this in the manuscript by revising the Evaluation design subsection within Modeling framework.

Overfitting control: We thank the reviewer for this comment. Several aspects of our modeling framework help mitigate overfitting. First, model performance is evaluated on held-out test data under the 80–20 split, ensuring that results reflect out-of-sample predictive ability. Second, the use of lagged predictors and a common modeling structure across approaches limits excessive model flexibility. Third, for machine learning models, we use standard implementations with moderate complexity which provide inherent safeguards against overfitting.

Finally, the rolling-window analyses further assess model stability across different temporal subsets, providing an additional check on overfitting. Taken together, these design choices help ensure that the reported results are not driven by overfitting.

Evaluation metrics: We thank the reviewer for this suggestion. In addition to the existing metrics, we have incorporated the mean absolute scaled error (MASE) as a scale-independent performance measure relative to a naive baseline. This provides a more robust assessment of predictive performance, particularly under potential level shifts. The manuscript has been updated accordingly.

Hyperparameter choices: We thank the reviewer for this important suggestion. To ensure a fair and reproducible comparison, all models were implemented using standard framework settings, consistent with common benchmarking practice. To assess sensitivity to hyperparameter choices, we conducted additional analyses using two alternative hyperparameter configurations (AHP1: Layer Configuration - (27, 9, 3); Epochs - 75; Learning Rate - 0.01; Momentum - 0.9; Alpha - 0.01; Validation Fraction - 0.25 and AHP2: Layer Configuration - (16, 8, 4, 2); Epochs - 150; Learning Rate - 0.001; Momentum - 0.8; Alpha - 0.001; Validation Fraction - 0.9). The results (shown in the Table R3 below) indicate that the impact of hyperparameter selection is model-dependent. In particular, SGD exhibits low variability across settings, Adam shows substantial sensitivity with frequent degradation in performance, and L-BFGS remains relatively stable under moderate changes (AHP1) but can deteriorate under more extreme configurations (AHP2). Importantly, these variations do not materially alter the overall conclusions regarding target-dependent predictability and the relative performance patterns across models. Based on these findings, we retain standard settings for the primary analysis to ensure comparability and reproducibility.

Table R3: Model performances under different hyperparameter choices

Hyper-parameter	Targets	Models	80 - 20			12 -- 4			6 -- 1		
			MAE	RMSE	MASE	MAE	RMSE	MASE	MAE	RMSE	MASE
Regular HP	EM.T	SGD	4459.52	4572.99	67.59	431.4	539.20	7.70	196.65	301.67	3.55
		Adam	16242.77	16542.75	246.20	12030.1	14336.72	214.82	12581.96	14842.12	227.43
		L-BFGS	3775.77	4093.48	57.23	478.27	933.38	8.54	244.7	421.03	4.42
	BA.T	SGD	4231.46	4986.33	5.43	1552.79	2818.38	1.87	1229.67	2312.91	1.49
		Adam	11186.67	11650.22	14.35	12968.64	14694.10	15.61	13518.6	15246.71	16.33
		L-BFGS	2376.28	3130.45	3.05	1237.11	2159.09	1.49	1277.44	2715.53	1.54
	CTS.T	SGD	18905.24	21295.07	26.75	2742.23	3551.82	4.14	1468.84	1922.94	2.21
		Adam	4184.56	4843.33	5.92	2050.68	2616.49	3.10	1222.21	1588.59	1.84
		L-BFGS	3140.23	3971.19	4.44	1539.46	2268.47	2.33	1096.82	1595.91	1.65
AHP 1	EM.T	SGD	3355.72	3501.28	50.81	406.83	517.38	7.28	196.89	299.37	3.57
		Adam	21007.06	21495.03	318.10	6448.81	7101.82	115.41	6483.61	6984.22	117.47
		L-BFGS	4691.75	4799.02	71.04	474.65	683.49	8.49	275.28	444.75	4.99
	BA.T	SGD	12017.16	12116.67	18.27	1071.07	1796.33	1.33	1419.9	2564.17	1.76
		Adam	13803.56	14473.00	20.98	6879.07	7523.89	8.52	7052.77	7642.99	8.76
		L-BFGS	1450.7	1744.16	2.21	1122.4	1850.72	1.39	1166.35	2394.06	1.45
	CTS.T	SGD	17129.56	18571.79	24.21	2499.48	3570.29	3.78	1472.85	1920.28	2.22
		Adam	5534.17	6212.96	7.82	4887.93	7163.21	7.39	4910.29	7200.85	7.39
		L-BFGS	4671.02	5071.25	6.60	1471.53	2116.88	2.22	1084.51	1626.85	1.63
AHP 2	EM.T	SGD	4073.87	4197.62	61.69	369.54	453.54	6.61	186.38	285.73	3.38
		Adam	25441.02	26468.80	385.24	19628.62	21011.74	351.27	18878.57	19296.63	342.03
		L-BFGS	21280.86	21304.90	322.24	416.61	632.72	7.46	363.47	1474.96	6.59
	BA.T	SGD	18596.08	19642.22	28.27	1184.17	1915.16	1.47	1168.78	2104.50	1.45
		Adam	30313.28	31244.96	46.08	19125.11	21023.46	23.69	18329.23	19322.82	22.77
		L-BFGS	26057.59	26103.72	39.61	990.19	1863.79	1.23	7744.5	91720.07	9.62
	CTS.T	SGD	25706.3	26768.60	36.33	2777.1	3849.60	4.20	1564.3	2170.07	2.35
		Adam	62240.7	62791.39	87.96	47624.08	48901.85	72.00	46630.44	47577.65	70.17
		L-BFGS	44225.63	48589.14	62.50	4206.3	10088.27	6.36	2207.24	5712.69	3.32

9. "Results: The results section communicates the main findings, and Table 1 provides a useful cross-target and cross-scheme comparison. The consistency of model rankings across the 80–20 and 12–4 settings is the section's strongest feature, but I would encourage the authors to revisit several claims that currently exceed the evidence. Statements such as reproducible predictive information, underlying signal rather than model-specific artifacts, and not overfitting require formal justification. I recommend that the authors supplement the reported point estimates and MAE confidence intervals with statistical comparisons of forecast accuracy across models, using the Diebold-Mariano test or an equivalent procedure, alongside explicit baseline skill scores

against seasonal-naive and autoregressive-only models. Without these, the conclusions are difficult to defend.

It is also advisable for the authors to qualify the characterization of CTS.T as more difficult. While this description may be appropriate under the 80–20 split, Random Forest and L-BFGS demonstrate competitive performance on CTS.T in the 12–4 scheme. A more accurate positioning would be that predictability depends on both the model and the experimental design, rather than being an inherent property of the target.

The figures present issues that should be addressed directly. Figure 3 reveals systematic errors at level changes and trend accelerations, while the trajectories in Figure 4 appear excessively smooth compared to the observed series. This pattern suggests underfitting or delayed model responsiveness rather than effective noise reduction. This distinction is important, as predictions that appear stable may fail at turning points, which are often the most policy-relevant periods.

Additionally, direct comparisons of MAE or RMSE across the 80–20 and 12–4 designs should be avoided, as the test windows differ in both length and likely difficulty.

Finally, I recommend that the authors include a brief methods note clarifying how the 95% confidence intervals for MAE were constructed, and specifying whether a bootstrap, an empirical window-level distribution, or an asymptotic approximation was used. This clarification is important because serial dependence can invalidate standard interval calculations. Thank you."

Response:

Overstated claims / lack of formal justification: We thank the reviewer for this important comment. We agree that some statements in the Results section overstated the level of formal support for the observed patterns. We have revised the text to adopt a more cautious interpretation, avoiding claims regarding reproducible signal, intrinsic structural relationships, or absence of overfitting. The revised wording emphasizes descriptive patterns in predictive performance across models and evaluation designs, without attributing these differences to underlying mechanisms or generalizable signal beyond the scope of the analysis.

Missing statistical comparison of models: We thank the reviewer for this valuable suggestion. We considered formal forecast comparison tests such as the Diebold–Mariano (DM) test but opted against them for concrete statistical and practical reasons. First, the rolling evaluation schemes — particularly the 6–1 and 12–4 designs — produce short test windows, under which DM tests are severely underpowered and may yield unreliable inference. Second, conducting pairwise comparisons across six models, three targets, and three evaluation schemes would generate a large number of simultaneous tests, requiring substantial multiple testing corrections that would further reduce power and complicate interpretation. Third, our primary objective is not to establish the formal superiority of any single model but to assess the consistency of predictive performance patterns across models and evaluation designs — a goal better served by reporting multiple complementary metrics across settings than by pairwise hypothesis testing.

Accordingly, we report MAE with bootstrap confidence intervals, RMSE, and MASE, and we have revised all related claims to reflect the descriptive nature of the comparisons. We have clarified this scope explicitly in the Performance Metrics subsection.

Accordingly, we add the following in the "Performance Metrics" subsection:

"*We do not conduct formal statistical tests of differences in forecast accuracy (e.g., Diebold–Mariano tests). The short test windows in the rolling schemes render such tests severely underpowered, and pairwise comparisons across six models, three targets, and three evaluation schemes would require substantial multiple testing corrections that complicate interpretation. The primary objective is descriptive comparison of performance patterns across models and evaluation frameworks rather than establishing formal model superiority.*"

<u>*CTS.T interpretation issue*</u>: We thank the reviewer for this important observation. We agree that the characterization of CTS.T as inherently more difficult to predict was too strong. We have revised the text to clarify that predictive performance for CTS.T varies across evaluation designs and modeling approaches. In particular, while CTS.T exhibited higher errors under the 80–20 split, several models (e.g., Random Forest and L-BFGS) demonstrated competitive performance under rolling-window schemes. The revised wording emphasizes that predictability depends on both the model and the evaluation design rather than being an intrinsic property of the target.

<u>*Figure interpretation (model behavior)*</u>: We thank the reviewer for this insightful comment. We agree that the prediction trajectories in Figures 3 and 4 exhibit smoothing relative to the observed series and may not fully capture rapid level changes or trend accelerations. We have revised the text to clarify that this behavior likely reflects limited responsiveness to abrupt changes, which may be interpreted as underfitting or lagged adjustment rather than effective noise reduction. This limitation is particularly relevant for forecasting turning points, which are often of practical importance.

We added the following in the Result section:

"*In addition, visual inspection of Figures 3 and 4 suggests that several models produce smoother trajectories than the observed series and may not fully capture abrupt level changes or periods of rapid trend acceleration. This pattern indicates limited responsiveness to short-term fluctuations, consistent with underfitting or lagged adjustment rather than effective noise reduction.*"

<u>*Invalid cross-design metric comparison*</u>: We thank the reviewer for this important clarification. We agree that direct comparisons of error metrics across evaluation designs should be made cautiously due to differences in test windows and underlying data difficulty. We have revised the text to soften such comparisons and to qualify them appropriately. For instance, where the manuscript notes that MAE values were lower in the 12–4 setting than in the 80–20 scheme, this is now framed as a descriptive observation about temporal localization rather than a claim of superior performance, and no strong inferential conclusions are drawn from it. Performance across evaluation frameworks is otherwise interpreted separately.

<u>*Unclear confidence interval construction*</u>: We thank the reviewer for this important clarification. The 95% confidence intervals for MAE were computed using a bootstrap procedure applied to

the test-period prediction errors. This approach accounts for variability in forecast errors without relying on asymptotic assumptions. We have added the following description of this procedure in the Methods section:

"*The 95% confidence intervals for MAE were obtained using a bootstrap procedure applied to the test-period prediction errors, which provides an empirical estimate of uncertainty and partially accounts for temporal dependence in the error structure.*"

General strengthening of evidence: We thank the reviewer for this important comment. We agree that stronger emphasis on the level of evidence is warranted. Accordingly, we have revised the Results and Discussion sections to adopt a more cautious interpretation of findings, focusing on descriptive comparisons of predictive performance across models and evaluation designs. We avoid statements implying formal statistical superiority or underlying structural relationships and instead emphasize consistency of observed patterns. We have also incorporated scale-independent metrics (MASE) and clarified the construction of confidence intervals to strengthen the empirical basis of the analysis.

10. "Discussion: The discussion and conclusion are aligned with the study's forecasting objective, appropriately avoid causal claims, and effectively highlight the practical relevance of model stability for health-system planning under economic volatility. I would commend the authors for their emphasis on predictive association over mechanism, the cross-framework comparison, and the deployment-oriented framing of reliability and transparency; these are genuine strengths of the section.

That said, I would encourage the authors to present claims regarding structural linkages, greater robustness, and models emphasizing stability and implicit regularization with greater caution. Unless directly supported by formal comparisons, such as baseline-relative skill scores, forecast comparison tests, or repeated-window rank stability summaries, these assertions risk overstating what the results demonstrate. I also suggest the authors explicitly acknowledge that observed differences across models may reflect tuning choices, window-length sensitivity, and data-regime dependence, rather than the intrinsic superiority of any particular optimization strategy.

The limitations section is comprehensive and appropriately addresses lag specification, selective indicators and targets, and the absence of feature attribution. I recommend that the authors add a sentence on real-time data availability and revision effects, as these are central to operational forecasting validity and were raised earlier in the methods discussion. This addition would bring the limitations into closer alignment with the methodological concerns flagged upstream.I believe that these comments can help improve the manuscript submission. Thank you."

Response:

Overstated claims: We thank the reviewer for this important comment. We agree that some statements in the Discussion section overstated the level of evidence, particularly regarding structural linkages, robustness, and model-specific properties. We have revised the Discussion to adopt more cautious language, avoiding claims that imply intrinsic relationships or model superiority. The revised text emphasizes that observed performance differences are descriptive and may depend on modeling choices, data characteristics, and evaluation design.

Missing formal support for claims: We thank the reviewer for this suggestion. We note that the objective of this study is descriptive comparison of predictive performance across models and evaluation designs rather than formal statistical comparison. Accordingly, we have revised the Discussion to avoid claims that would require formal hypothesis testing or baseline-relative skill measures, and instead focus on consistent patterns observed across settings.

Overinterpretation of model superiority: We thank the reviewer for this comment. We agree that differences in model performance should not be interpreted as evidence of intrinsic superiority. We have revised the Discussion to clarify that observed differences may reflect model specification, tuning choices, training window length, and data characteristics rather than inherent advantages of any particular modeling approach. We have added the following in the Discussion:

"*Observed differences in model performance should be interpreted cautiously, as they may reflect model specification, tuning choices, training window length, data-regime dependence, and other data characteristics rather than intrinsic superiority of any specific modeling approach.*"

Need for explicit acknowledgment of sensitivity: We thank the reviewer for this comment. We have revised the Discussion to explicitly acknowledge that model performance may be sensitive to modeling choices, training window length, and temporal data structure, and that observed differences should be interpreted within this context.

Limitation gap: We thank the reviewer for this suggestion. We have added a statement in the limitations section noting that the analysis is based on revised data and does not explicitly account for real-time data availability or revision effects, which may affect operational forecasting performance:

"*An additional limitation is that the analysis is based on revised data and does not explicitly account for real-time data availability or subsequent data revisions, which may influence forecasting performance in operational settings.*"

Editorial and Technical Revisions

In addition to the reviewer comments, we have also addressed the journal's technical requirements:

- Figures have been uploaded as separate high-resolution eps files.
- Legends for Supporting Information files have been added.
- The Data Availability Statement has been added to include dataset access details.

We thank the editor and reviewers again for their constructive feedback and believe that the revisions have substantially improved the clarity, transparency, and rigor of the manuscript.

Sincerely,

Soutik Ghosal, PhD

(on behalf of all authors)
