# Supplementary material for: Assessing the informative value of macroeconomic indicators for public health forecasting
Source: PLOS Digit Health. 2026 Aug 20;5(8):e0001599. doi: 10.1371/journal.pdig.0001599 (PMC13492747; doi:10.1371/journal.pdig.0001599)
Supplement: Peer review history

# Peer review: Decision Letter 0

**DOI:** 10.1371/journal.pdig.0001599.r001

**Authors:** Dukyong Yoon, Peng Luo

26 Feb 2026

PDIG-D-26-00132

Assessing the informative value of macroeconomic indicators for public health forecasting

PLOS Digital Health

Dear Dr. Ghosal,

Thank you for submitting your manuscript to PLOS Digital Health. After careful consideration, we feel that it has merit but does not fully meet PLOS Digital Health's publication criteria as it currently stands. Therefore, we invite you to submit a revised version of the manuscript that addresses the points raised during the review process.

Please submit your revised manuscript by Mar 28 2026 11:59PM. If you will need more time than this to complete your revisions, please reply to this message or contact the journal office at digitalhealth@plos.org. When you're ready to submit your revision, log on to https://www.editorialmanager.com/pdig/ and select the 'Submissions Needing Revision' folder to locate your manuscript file.

Please include the following items when submitting your revised manuscript:

* A letter that responds to each point raised by the editor and reviewer(s). You should upload this letter as a separate file labeled 'Response to Reviewers'. This file does not need to include responses to any formatting updates and technical items listed in the 'Journal Requirements' section below.

* A marked-up copy of your manuscript that highlights changes made to the original version. You should upload this as a separate file labeled 'Revised Manuscript with Track Changes'.

* An unmarked version of your revised paper without tracked changes. You should upload this as a separate file labeled 'Manuscript'.

If you would like to make changes to your financial disclosure, competing interests statement, or data availability statement, please make these updates within the submission form at the time of resubmission. Guidelines for resubmitting your figure files are available below the reviewer comments at the end of this letter.

We look forward to receiving your revised manuscript.

Kind regards,

Peng Luo

Academic Editor

PLOS Digital Health

Dukyong Yoon

Section Editor

PLOS Digital Health

Leo Anthony Celi

Editor-in-Chief

PLOS Digital Health

orcid.org/0000-0001-6712-6626

**Journal Requirements:**

1. We ask that a manuscript source file is provided at Revision. Please upload your manuscript file as a .doc, .docx, .rtf or .tex.

2. Please upload separate figure files in .tif or .eps format. Also, remove the figures from your manuscript file but keep the legends.

For more information about figure files please see our guidelines:

https://journals.plos.org/digitalhealth/s/figures

https://journals.plos.org/digitalhealth/s/figures#loc-file-requirements

3. We have noticed that you have uploaded Supporting Information files, but you have not included a list of legends. Please add a full list of legends for your Supporting Information files after the references list.

4. Please note that your Data Availability Statement is currently missing the DOI/accession number of each dataset OR a direct link to access each database. If your manuscript is accepted for publication, you will be asked to provide these details on a very short timeline. We therefore suggest that you provide this information now, though we will not hold up the peer review process if you are unable.

If the reviewer comments include a recommendation to cite specific previously published works, please review and evaluate these publications to determine whether they are relevant and should be cited. There is no requirement to cite these works unless the editor has indicated otherwise.

**Additional Editor Comments (if provided):**

[Note: HTML markup is below. Please do not edit.]

**Reviewers' Comments:**

Reviewer's Responses to Questions

**Comments to the Author**

1. Does this manuscript meet PLOS Digital Health’s publication criteria? Is the manuscript technically sound, and do the data support the conclusions? The manuscript must describe methodologically and ethically rigorous research with conclusions that are appropriately drawn based on the data presented.

Reviewer #1: Yes

Reviewer #2: Yes

**********

2. Has the statistical analysis been performed appropriately and rigorously?

Reviewer #1: Yes

Reviewer #2: No

**********

3. Have the authors made all data underlying the findings in their manuscript fully available (please refer to the Data Availability Statement at the start of the manuscript PDF file)?

The PLOS Data policy requires authors to make all data underlying the findings described in their manuscript fully available without restriction, with rare exception. The data should be provided as part of the manuscript or its supporting information, or deposited to a public repository. For example, in addition to summary statistics, the data points behind means, medians and variance measures should be available. If there are restrictions on publicly sharing data—e.g. participant privacy or use of data from a third party—those must be specified.

Reviewer #1: Yes

Reviewer #2: Yes

**********

4. Is the manuscript presented in an intelligible fashion and written in standard English?

PLOS Digital Health does not copyedit accepted manuscripts, so the language in submitted articles must be clear, correct, and unambiguous. Any typographical or grammatical errors should be corrected at revision, so please note any specific errors here.

Reviewer #1: Yes

Reviewer #2: Yes

**********

5. Review Comments to the Author

Please use the space provided to explain your answers to the questions above. You may also include additional comments for the author, including concerns about dual publication, research ethics, or publication ethics. (Please upload your review as an attachment if it exceeds 20,000 characters)

Reviewer #1: 1. The authors should provide detailed information on the neural network architectures, including the number of layers and the activation functions used, as well as the optimization strategies.

2. To support their claim that these indicators are "leading signals," the authors should conduct cross-correlation analysis or use AIC/BIC to find the most predictive time lags for each indicator.

3. The authors need to explain how the models were recalibrated for "Period 4" (COVID-19), since this period likely disrupted previous weightings.

4. In the Methodology section, specifically in the overview of modeling approaches.It is suggested to refer from the paper: “ A Comprehensive Review of Artificial Intelligence Models for Lifetime Value Optimization “. This reference provides a useful comparison for probabilistic and machine learning models. Since the manuscript looks at different modeling methods for long-term health system targets, this citation supports the choice of ensemble and deep learning methods over deterministic ones.

5. The authors should clarify whether they used any dimensionality reduction methods, such as PCA, before fitting models to address correlated economic features.

6. The authors should include a table that defines each acronym, such as BA.F and IN.F, to help non-economist readers.

Reviewer #2: REVIEW

Abstract

This abstract addresses a significant question in public health forecasting and planning, and the research is clearly presented. Comparing various forecasting methods for capacity targets is a notable strength. To further strengthen the statistical analysis, briefly outline your evaluation framework, specify forecast horizons, model validation approaches, and performance metrics. Please also clarify how you assessed the predictive value of macroeconomic indicators beyond trends and seasonality. Including a brief explanation of how you evaluated model stability during periods of economic fluctuation will enhance the credibility of your conclusions. Thank you.

Introduction

The introduction provides a strong rationale for the study, supported by a comprehensive literature review and a clearly articulated research question. To enhance clarity, I encourage the authors to define the public health targets and capacity measures at the outset and to briefly justify their selection as proxies for health system strain.

From a methodological perspective, the introduction should show greater specificity regarding the assessment of informative value. In particular, it should clarify whether the analysis examines incremental predictive value beyond trend and seasonality, delineate the role of baseline time-series models, and specify the approach to forecast performance validation, such as a fixed split, a rolling window, or both. Providing these details is essential to support the paper's contribution and to ensure alignment between the introduction and the subsequennt analytical claims. Thank you.

Methods

The methods section is robust and reproducible; however, there are several aspects that I think require clarification. While the one-month lag is appropriate, the manuscript should explicitly discuss real-time data availability, publication lags, and revision histories for both predictors and targets. Employing revised or seasonally adjusted series without specifying the information available at each time point may artificially enhance perceived predictabilityy.

The figures show clear structural breaks at the Great Reccession and the COVID-19 pandemic, yet the manuscript does not explain how nonstationarity and regime change are handled. ARIMAX requires explicit justification of differencing and seasonal term selection; ML models can inadvertently capture trend without explicit time features or de-trending. The central claim, that macroeconomic indicators improve forecast accuracy, also requires a clean baseline comparison: each model should have a counterpart using only autoregressive terms, with economic indicators added incrementally. Without this, the analysis cannot isolate the contribution of macroeconomic variables from the series' inherent extrapolabilityy.

The 80–20 data split is appropriate; however, the rolling schemes (12–4 and 6–1) are unconventional and likely underpowered, especially for neural networks and random forests. The manuscript should justify the use of short training windows and detail the methods employed to control overfitting. An expanding-window rolling-origin evaluation would provide a more defensible approach and better reflect operational practice. Regarding evaluation metrics, N-RMSE normalized by the test mean is unstable under level shifts; MASE or skill scores relative to a seasonal-naive or autoregressive baseline would offer a more rigorous assessment.

Default hyperparameters are insufficiently justified unless their suitability for this specific problem is explicitly shown. Limited, pre-specified tuning within appropriate time-series resampling frameworks is preferable. At a minimum, sensitivity analyses should confirm that the conclusions are not dependent on arbitrary default settings.. These suggestions should improve the methds section. Thank you.

Results

The results section communicates the main findings, and Table 1 provides a useful cross-target and cross-scheme comparison. The consistency of model rankings across the 80–20 and 12–4 settings is the section's strongest feature, but I would encourage the authors to revisit several claims that currently exceed the evidence. Statements such as reproducible predictive information, underlying signal rather than model-specific artifacts, and not overfitting require formal justification. I recommend that the authors supplement the reported point estimates and MAE confidence intervals with statistical comparisons of forecast accuracy across models, using the Diebold-Mariano test or an equivalent procedure, alongside explicit baseline skill scores against seasonal-naive and autoregressive-only models. Without these, the conclusions are difficult to defend.

It is also advisable for the authors to qualify the characterization of CTS.T as more difficult. While this description may be appropriate under the 80–20 split, Random Forest and L-BFGS demonstrate competitive performance on CTS.T in the 12–4 scheme. A more accurate positioning would be that predictability depends on both the model and the experimental design, rather than being an inherent property of the target.

The figures present issues that should be addressed directly. Figure 3 reveals systematic errors at level changes and trend accelerations, while the trajectories in Figure 4 appear excessively smooth compared to the observed series. This pattern suggests underfitting or delayed model responsiveness rather than effective noise reduction. This distinction is important, as predictions that appear stable may fail at turning points, which are often the most policy-relevant periods. Additionally, direct comparisons of MAE or RMSE across the 80–20 and 12–4 designs should be avoided, as the test windows differ in both length and likely difficulty.

Finally, I recommendd that the authors include a brief methods note clarifying how the 95% confidence intervals for MAE were constructed, and specifying whether a bootstrap, an empirical window-level distribution, or an asymptotic approximation was used. This clarification is important because serial dependence can invalidate standard interval calculations. Thank you.

Discussion and Conclusion

The discussion and conclusion are aligned with the study's forecasting objective, appropriately avoid causal claims, and effectively highlight the practical relevance of model stability for health-system planning under economic volatility. I would commend the authors for their emphasis on predictive association over mechanism, the cross-framework comparison, and the deployment-oriented framing of reliability and transparency; these are genuine strengths of the section.

That said, I would encourage the authors to present claims regarding structural linkages, greater robustness, and models emphasizing stability and implicit regularization with greater caution. Unless directly supported by formal comparisons, such as baseline-relative skill scores, forecast comparison tests, or repeated-window rank stability summaries, these assertions risk overstating what the results demonstrate. I also suggest the authors explicitly acknowledge that observed differences across models may reflect tuning choices, window-length sensitivity, and data-regime dependence, rather than the intrinsic superiority of any particular optimization strategy.

The limitations section is comprehensive and appropriately addresses lag specification, selective indicators and targets, and the absence of feature attribution. I recommend that the authors add a sentence on real-time data availability and revision effects, as these are central to operational forecasting validity and were raised earlier in the methods discussion. This addition would bring the limitations into closer alignment with the methodological concerns flagged upstream.

I believe that these comments can help improve the manuscript submission. Thank you.

**********

6. PLOS authors have the option to publish the peer review history of their article (what does this mean?). If published, this will include your full peer review and any attached files.

**Do you want your identity to be public for this peer review?**  If you choose “no”, your identity will remain anonymous but your review may still be made public.

For information about this choice, including consent withdrawal, please see our Privacy Policy.

Reviewer #1: No

Reviewer #2: **Yes:** Victor Ifechukwude Agboli

**********

[NOTE: If reviewer comments were submitted as an attachment file, they will be attached to this email and accessible via the submission site. Please log into your account, locate the manuscript record, and check for the action link "View Attachments". If this link does not appear, there are no attachment files.]

**Figure resubmission:**

While revising your submission, we strongly recommend that you use PLOS’s NAAS tool (https://ngplosjournals.pagemajik.ai/artanalysis) to test your figure files. NAAS can convert your figure files to the TIFF file type and meet basic requirements (such as print size, resolution), or provide you with a report on issues that do not meet our requirements and that NAAS cannot fix.

After uploading your figures to PLOS’s NAAS tool - https://ngplosjournals.pagemajik.ai/artanalysis, NAAS will process the files provided and display the results in the "Uploaded Files" section of the page as the processing is complete. If the uploaded figures meet our requirements (or NAAS is able to fix the files to meet our requirements), the figure will be marked as "fixed" above. If NAAS is unable to fix the files, a red "failed" label will appear above. When NAAS has confirmed that the figure files meet our requirements, please download the file via the download option, and include these NAAS processed figure files when submitting your revised manuscript.

**Reproducibility:**

To enhance the reproducibility of your results, we recommend that authors of applicable studies deposit laboratory protocols in protocols.io, where a protocol can be assigned its own identifier (DOI) such that it can be cited independently in the future. Additionally, PLOS ONE offers an option to publish peer-reviewed clinical study protocols. Read more information on sharing protocols at https://plos.org/protocols?utm_medium=editorial-email&utm_source=authorletters&utm_campaign=protocols

# Author comment: Author response to Decision Letter 1

**DOI:** 10.1371/journal.pdig.0001599.r002

11 May 2026

Attachment Submitted filename: Response to Reviewers.pdf

# Editor report: Decision Letter 1

**DOI:** 10.1371/journal.pdig.0001599.r003

**Authors:** Dukyong Yoon

5 Jul 2026

Assessing the informative value of macroeconomic indicators for public health forecasting

PDIG-D-26-00132R1

Dear Mr. Ghosal,

We are pleased to inform you that your manuscript 'Assessing the informative value of macroeconomic indicators for public health forecasting' has been provisionally accepted for publication in PLOS Digital Health.

Before your manuscript can be formally accepted you will need to complete some formatting changes, which you will receive in a follow-up email from a member of our team.

Please note that your manuscript will not be scheduled for publication until you have made the required changes, so a swift response is appreciated.

IMPORTANT: The editorial review process is now complete. PLOS will only permit corrections to spelling, formatting or significant scientific errors from this point onwards. Requests for major changes, or any which affect the scientific understanding of your work, will cause delays to the publication date of your manuscript.

If your institution or institutions have a press office, please notify them about your upcoming paper to help maximize its impact. If they'll be preparing press materials, please inform our press team as soon as possible -- no later than 48 hours after receiving the formal acceptance. Your manuscript will remain under strict press embargo until 2 pm Eastern Time on the date of publication. For more information, please contact digitalhealth@plos.org.

Thank you again for supporting Open Access publishing; we are looking forward to publishing your work in PLOS Digital Health.

Best regards,

Dukyong Yoon

Section Editor

PLOS Digital Health

***********************************************************

**Additional Editor Comments (if provided):**

**Reviewer Comments (if any, and for reference):**
